# Fear, anxiety, and the extended amygdala—*Absence of evidence for strict functional segregation*

**DOI:** 10.1101/2025.08.29.673083

**Published:** 2025-09-04

**Authors:** Paige R. Didier, Shannon E. Grogans, Claire M. Kaplan, Hyung Cho Kim, Samiha Islam, Allegra S. Anderson, Rachael M. Tillman, Manuel Kuhn, Juyoen Hur, Andrew S. Fox, Kathryn A. DeYoung, Jason F. Smith, Alexander J. Shackman

**Affiliations:** 1 Department of Psychology, University of Maryland, College Park, MD 20742 USA.; 2 Neuroscience and Cognitive Science Program, University of Maryland, College Park, MD 20742 USA.; 3 Maryland Neuroimaging Center, University of Maryland, College Park, MD 20742 USA.; 4 Department of Psychiatry and Behavioral Sciences, School of Medicine, Johns Hopkins University, Baltimore, MD 21205 USA.; 5 Department of Psychology, University of Pennsylvania, Philadelphia, PA USA.; 6 Department of Psychiatry and Human Behavior, Brown University, Providence, RI 02912 USA.; 7 McGill Neuropsychology, Bethesda, MD 20814 USA.; 8 Center for Depression, Anxiety and Stress Research, McLean Hospital, Harvard Medical School, Belmont, MA 02478 USA.; 9 Department of Psychology, Yonsei University, Seoul 03722, Republic of Korea.; 10 Department of Psychology, University of California, Davis, CA 95616 USA; 11 California National Primate Research Center, University of California, Davis, CA 95616 USA

**Keywords:** affective neuroscience, fear and anxiety, bed nucleus of the stria terminalis (BST/BNST), extended amygdala (EA), Research Domain Criteria (RDoC)

## Abstract

Since the time of Freud, the distinction between fear and anxiety has been a hallmark of influential models of emotion and emotional illness, including the Diagnostic and Statistical Manual of Mental Disorders (DSM) and Research Domain Criteria (RDoC) framework. Fear and anxiety disorders are a leading cause of human misery and morbidity. Existing treatments are inconsistently effective, underscoring the importance of developing accurate models of the underlying neurobiology. Although there is agreement that the extended amygdala (EA) plays a central role in orchestrating responses to threat, the respective contributions of its two major subdivisions—the central nucleus of the amygdala (Ce) and bed nucleus of the stria terminalis (BST)—remain contentious. To help adjudicate this debate, we performed a harmonized mega-analysis of fMRI data acquired from 295 adults as they completed a well-established threat-anticipation paradigm. Contrary to popular double-dissociation models, results demonstrated that the Ce responds to temporally uncertain threat and the BST responds to certain threat. In direct comparisons, the two regions showed statistically indistinguishable responses, with strong Bayesian evidence of regional equivalence. These observations underscore the need to reformulate conceptual models that posit a strict segregation of temporally certain and uncertain threat processing in the EA.

## INTRODUCTION

Since the time of Freud, the distinction between fear and anxiety has been a hallmark of influential models of emotion and emotional illness, including the DSM and Research Domain Criteria (RDoC) framework (APA, 2022; Freud et al., 1959; Grogans et al., 2023; Grupe & Nitschke, 2013; LeDoux & Pine, 2016; Mobbs et al., 2019; NIMH, 2011; Tovote et al., 2015). When extreme or pervasive, fear and anxiety can become debilitating (Salomon et al., 2015). Anxiety disorders are a leading cause of human misery, morbidity, and premature mortality (GBD, 2024; Plana-Ripoll et al., 2020; Weye et al., 2020). Existing treatments are far from curative for many, underscoring the need to develop a more complete and accurate understanding of the underlying neurobiology (Batelaan et al., 2017; Cuijpers et al., 2024; Singewald et al., 2023; Strawn et al., 2021).

There is widespread consensus that the extended amygdala (EA)—a macrocircuit encompassing the central nucleus of the amygdala (Ce) and bed nucleus of the stria terminalis (BST)—plays a central role in fear and anxiety-related states, traits, and disorders, but the precise contributions of the Ce and BST remain contentious (Blanchard & Canteras, 2024; Daniel-Watanabe & Fletcher, 2022; Hur et al., 2020; Shackman et al., 2024). RDoC and other double-dissociation models organize fear and anxiety into two strictly segregated neural systems (Supplementary Figure S1 and Note 1): the *Acute Threat* system is centered on the amygdala (including the Ce), is sensitive to certain (but not uncertain) threat, and promotes signs and symptoms of fear; whereas the *Potential Threat* system is centered on the BST, is sensitive to uncertain (but not certain) threat, and promotes anxiety (Avery et al., 2016; LeDoux & Pine, 2016; NIMH, 2011). Yet a growing body of rodent mechanistic data casts doubt on this either/or perspective (Ahrens et al., 2018; Bruzsik et al., 2021; Chen et al., 2022; Griessner et al., 2021; Gungor & Paré, 2016; Holley & Fox, 2022; Lange et al., 2017; Marcinkiewcz et al., 2016; Marvar et al., 2021; Moscarello & Penzo, 2022; Pomrenze, Giovanetti, et al., 2019; Pomrenze, Tovar-Diaz, et al., 2019; Ren et al., 2022; Ressler et al., 2020; Zhu et al., 2024), motivating the competing hypothesis that the Ce and BST play a role in organizing responses to both kinds of threat (Daniel-Watanabe & Fletcher, 2022; Fox & Shackman, 2019; Gungor & Paré, 2016; Shackman & Fox, 2016).

**Figure 1.**
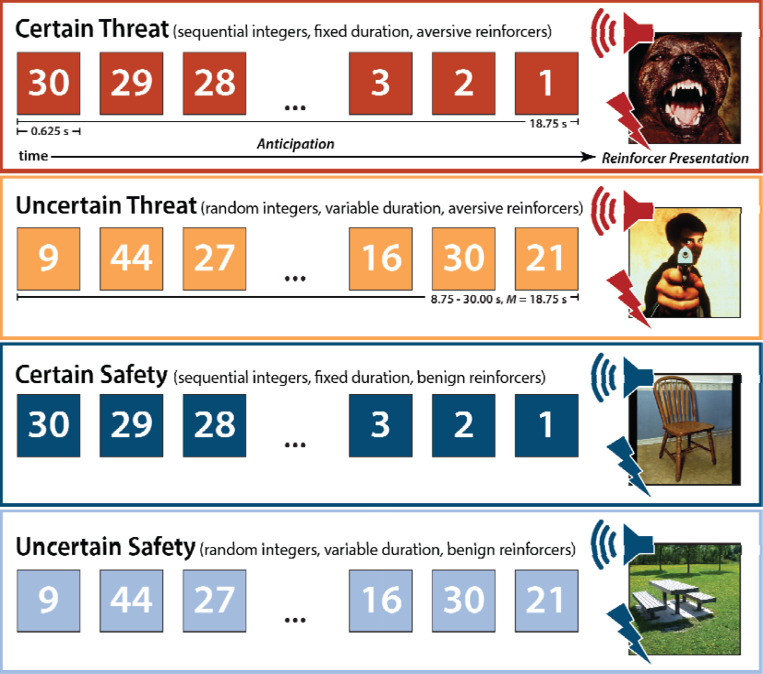
Maryland Threat Countdown fMRI Paradigm. The paradigm takes the form of a 2 (*Valence*: Threat, Safety) × 2 (*Temporal Certainty*: Certain, Uncertain) randomized event-related design. Participants were completely informed about the task design and contingencies prior to scanning. On certain-threat trials, participants saw a descending stream of integers (‘countdown’) for 18.75 s. To ensure robust emotion induction, the anticipation epoch always terminated with the presentation of a noxious electric shock, unpleasant photograph, and thematically related audio clip (e.g., scream). Uncertain-threat trials were similar, but the integer stream was randomized and presented for an uncertain and variable duration (8.75–30.00 s; *M*=18.75 s). Participants knew that something aversive was going to occur, but they had no way of knowing precisely *when*. Safety trials were similar but terminated with the delivery of emotionally neutral reinforcers (e.g., just-perceptible electrical stimulation). Abbreviation—s, seconds.

To help adjudicate this debate, we performed a harmonized mega-analysis of fMRI data acquired from 295 racially diverse adults as they completed the Maryland Threat Countdown (MTC), a well-established threat-anticipation paradigm (Figure 1) (Grogans et al., 2024; Hur et al., 2020; Kim et al., 2023). The MTC is an fMRI-optimized variant of temporally certain/uncertain-threat assays that have been behaviorally and pharmacologically validated in rodents and humans (Daldrup et al., 2015; Hefner et al., 2013; Lange et al., 2017; Miles et al., 2011; Moberg et al., 2017), maximizing translational relevance. Data were acquired using a multiband sequence and re-processed using a singular best-practices pipeline. The relatively large sample afforded the power necessary to reliably detect small differences in regional responses to certain- and uncertain-threat anticipation (*d*≥0.16).

Because voxelwise analyses do not permit inferences about regional differences, we used *a priori* anatomical regions of interest (ROIs) to rigorously compare the Ce and BST. This approach has the advantage of providing statistically unbiased effect-size estimates (Poldrack et al., 2017), in contrast to earlier work by our group that relied on functionally defined ROIs (Hur et al., 2020). To maximize anatomical resolution and inferential clarity, mean activation was computed using spatially unsmoothed data (Tillman et al., 2018). Hypothesis testing focused on regional responses to certain- and uncertain-threat anticipation relative to their perceptually similar reference conditions (e.g., certain-safety anticipation), providing sharper inferences than work focused on baseline contrasts (Grogans et al., 2024). Of course, traditional null-hypothesis tests cannot address whether the Ce and BST show statistically equivalent responses to certain- and uncertain-threat anticipation. Here we used a Bayesian framework to quantify the relative strength of the evidence for and against regional equivalence. The Bayesian approach provides well-established benchmarks for interpreting effect sizes and sidesteps the need to arbitrarily choose what constitutes a ‘statistically indistinguishable’ difference (Bo et al., 2024; van Doorn et al., 2021; E. J. Wagenmakers et al., 2018), in contrast to work that relied on frequentist equivalence tests (Shackman et al., 2024). Whole-brain voxelwise analyses enabled us to explore the relevance of other, less intensively scrutinized regions.

## METHOD

### Overview of the Mega-Analysis

The neuroimaging mega-analysis capitalized on data from two previously published fMRI studies focused on the neural circuits recruited by temporally certain- and uncertain-threat anticipation. The first study encompassed a sample of 220 psychiatrically healthy, first-year university students (Grogans et al., 2024). The second encompassed 75 tobacco smokers recruited from the surrounding community (Kim et al., 2023). Both studies employed the same certain/uncertain threat-anticipation paradigm (Maryland Threat Countdown task) and were collected using identical parameters on the same scanner using the same head-coil. For the mega-analysis, all neuroimaging data were completely reprocessed using a singular best-practices pipeline, as detailed below. All participants provided informed written consent. Procedures were approved by the University of Maryland, College Park Institutional Review Board (protocols #659385 and #824438).

Detailed descriptions of the study designs, enrollment criteria, participants, data collection procedures, and data exclusions are provided in the original reports (Grogans et al., 2024; Kim et al., 2023). The mega-analysis was not pre-registered.

Processed data, anatomical regions-of-interest, statistical code, and detailed results are available (https://osf.io/fcvdj). Task materials (https://osf.io/e2ngf) and key neuroimaging maps are also available (https://neurovault.org/collections/16083).

### Participants

Across studies, a racially diverse sample of 295 participants provided usable neuroimaging data (45.4% female; 52.2% White Nonhispanic, 16.6% Asian, 19.0% African American, 4.1% Hispanic, 8.1% Multiracial/Other; *M*=21.6 years, *SD*=5.7). Of these, 8 participants were excluded from skin conductance analyses due to insufficient usable data.

### Power Analysis

To enable readers to better interpret in our results, we performed a post hoc power analysis. G-power (version 3.1.9.2) indicated that the final sample of 295 usable fMRI datasets provides 80% power to detect ‘small’ mean differences in regional activation (Cohen’s *d*=0.16, *α*=0.05, two-tailed) (Cohen, 1988; Faul et al., 2007).

### Threat-Anticipation Paradigm

#### Paradigm Structure and Design Considerations.

The Maryland Threat Countdown paradigm is a well-established, fMRI-optimized variant of temporally uncertain-threat assays that have been validated in rodents and humans (Daldrup et al., 2015; Hefner et al., 2013; Lange et al., 2017; Miles et al., 2011; Moberg et al., 2017).

As shown schematically in Figure 1, the paradigm takes the form of a 2 (*Valence*: Threat/Safety) × 2 (*Temporal Certainty*: Uncertain/Certain) randomized, event-related, repeated-measures design (3 scans; 6 trials/condition/scan). Participants were completely informed about the task design and contingencies prior to scanning. Simulations were used to optimize the detection and deconvolution of task-related hemodynamic signals. Stimulus presentation was controlled using Presentation software (version 19.0, Neurobehavioral Systems, Berkeley, CA).

On Certain-Threat trials, participants saw a descending stream of integers (‘count-down;’ e.g., 30, 29, 28...3, 2, 1) for 18.75 s. To ensure robust distress and arousal, the anticipation epoch culminated with the presentation of a noxious electric shock, unpleasant photograph (e.g., mutilated body), and thematically related audio clip (e.g., gunshot). Uncertain-Threat trials were similar, but the integer stream was randomized and presented for an uncertain and variable duration (8.75–30.00 s; *M*=18.75 s). Participants knew that something aversive was going to occur but had no way of knowing precisely when. Consistent with methodological recommendations (Shackman & Fox, 2016), the mean duration of the anticipation epoch was identical across conditions, ensuring equal measurement precision. The specific duration was chosen to enhance detection of task-related differences in the blood oxygen level-dependent (BOLD) signal (‘activation’) (Henson, 2007) and to allow sufficient time for sustained responses to become evident. Safety trials were similar but terminated with the delivery of emotionally neutral reinforcers (see below). Valence was continuously signaled during the anticipation epoch (‘countdown’) by the background color of the display. Temporal certainty was signaled by the nature of the integer stream. Certain trials always began with the presentation of the number 30. On Uncertain trials, integers were randomly drawn from a near-uniform distribution ranging from 1 to 45 to reinforce the impression that they could be much shorter or longer than Certain trials and to minimize incidental temporal learning (‘time-keeping’). To concretely demonstrate the variable duration of Uncertain trials, during scanning, the first three Uncertain trials featured short (8.75 s), medium (15.00 s), and long (28.75 s) anticipation epochs. To mitigate potential confusion and eliminate mnemonic demands, a lower-case ‘c’ or ‘u’ was presented at the lower edge of the display throughout the anticipatory epoch. White-noise visual masks (3.2 s) were presented between trials to minimize the persistence of visual reinforcers in iconic memory.

Participants were periodically prompted (following the offset of the white-noise visual mask) to rate the intensity of fear/anxiety experienced a few seconds earlier, during the anticipation period of the prior trial, using a 1 (*minimal*) to 4 (*maximal*) scale and an MRI-compatible response pad (MRA, Washington, PA). Each condition was rated once per scan (16.7% trials). Skin conductance was continuously acquired throughout.

#### Procedures.

Prior to scanning, participants practiced an abbreviated version of the paradigm (without electrical stimulation) until they indicated and staff confirmed understanding. Benign and aversive electrical stimulation levels were individually titrated. *Benign Stimulation*. Participants were asked whether they could “reliably detect” a 20 V stimulus and whether it was “at all unpleasant.” If the participant could not detect the stimulus, the voltage was increased by 4 V and the process repeated. If the participant indicated that the stimulus was unpleasant, the voltage was reduced by 4 V and the process was repeated. The final level chosen served as the benign electrical stimulation during the imaging assessment. *Aversive Stimulation*. Participants received a 100 V stimulus and were asked whether it was “as unpleasant as you are willing to tolerate”—an instruction specifically chosen to maximize anticipatory distress and arousal. If the participant indicated that they were willing to tolerate more intense stimulation, the voltage was increased by 10 V and the process repeated. If the participant indicated that the stimulus was too intense, the voltage was reduced by 5 V and the process repeated. The final level chosen served as the aversive electrical stimulation during the imaging assessment. Following each scan, staff re-assessed whether stimulation was sufficiently intense and increased the level as necessary.

#### Electrical Stimuli.

Electrical stimuli (100 ms; 2 ms pulses every 10 ms) were generated using an MRI-compatible constant-voltage stimulator system (STMEPM-MRI; Biopac Systems, Inc., Goleta, CA) and delivered using MRI-compatible, disposable carbon electrodes (Biopac) attached to the fourth and fifth digits of the left hand.

#### Visual Stimuli.

Seventy-two aversive and benign photographs (1.8 s) were selected from the International Affective Picture System (for details, see Hur et al., 2020). Visual stimuli were back-projected (Powerlite Pro G5550, Epson America, Inc., Long Beach, CA) onto a semi-opaque screen mounted at the head-end of the scanner bore and viewed using a mirror mounted on the head-coil.

#### Auditory Stimuli.

Seventy-two aversive and benign auditory stimuli (0.8 s) were adapted from open-access online sources and delivered using an amplifier (PA-1 Whirlwind) with in-line noise-reducing filters and ear buds (S14; Sensimetrics, Gloucester, MA) fitted with noise-reducing ear plugs (Hearing Components, Inc., St. Paul, MN).

#### Skin Conductance.

Skin conductance was continuously acquired during each scan using a Biopac system (MP-150; Biopac Systems, Inc., Goleta, CA). Skin conductance (250 Hz; 0.05 Hz high-pass) was measured using MRI-compatible disposable electrodes (EL507) attached to the second and third digits of the left hand.

### MRI Data Acquisition

Data were acquired using a single Siemens Magnetom TIM Trio 3 Tesla scanner (32-channel head-coil). Foam inserts were used to immobilize the participant’s head within the head-coil. Participants were continuously monitored using an eye-tracker (Eyelink 1000; SR Research, Ottawa, Ontario, Canada) and the AFNI real-time motion plugin (Cox, 1996). Eye-tracking data were not recorded. Sagittal T1-weighted anatomical images were acquired using a magnetization prepared rapid acquisition gradient echo sequence (TR=2,400 ms; TE=2.01 ms; inversion time=1,060 ms; flip=8°; slice thickness=0.8 mm; in-plane=0.8×0.8 mm; matrix=300×320; field-of-view=240×256). A T2-weighted image was collected co-planar to the T1-weighted image (TR=3,200 ms; TE=564 ms; flip angle=120°). A multi-band sequence was used to collect oblique-axial echo-planar imaging (EPI) volumes (multiband acceleration=6; TR=1,250 ms; TE=39.4 ms; flip=36.4°; slice thickness=2.2 mm, number of slices=60; in-plane resolution=2.1875×2.1875 mm; matrix=96×96). Data were collected in the oblique-axial plane (approximately −20° relative to the AC-PC plane) to minimize susceptibility artifacts. Three 478-volume EPI scans were acquired. The scanner automatically discarded the first 7 volumes. To enable fieldmap correction, two oblique-axial spin echo (SE) images were collected in opposing phase-encoding directions (rostral-to-caudal and caudal-to-rostral) at the same location and resolution as the functional volumes (i.e., co-planar; TR=7,220 ms; TE=73 ms). Respiration and pulse were continuously acquired during scanning using a respiration belt and photo-plethysmograph affixed to the first digit of the non-dominant hand. Following the last scan, participants were removed from the scanner, debriefed, compensated, and discharged.

### Skin Conductance Data Processing Pipeline

Skin conductance data were processed using *PsPM* (version 4.0.2) and in-house Matlab (version 9.9.0.1467703) code (Bach et al., 2018; Bach & Friston, 2013). Data were orthogonalized with respect to pulse and respiration signals and de-spiked using *filloutliers* (150-sample moving-median widow; modified Akima cubic Hermite interpolation). Each scan was then band-pass filtered (0.009–0.333 Hz), median centered, and down-sampled (4 Hz). Participant-specific skin conductance response functions (SCRFs) were estimated by fitting the four parameters of the canonical SCRF (Bach et al., 2010) to the grand-average reinforcer response using *fmincon* and a cost function that maximized variance explained and penalized negative coefficients.

### MRI Pipeline

Methods were optimized to minimize spatial normalization error and other potential sources of noise and are similar to other recent work by our group (Cornwell et al., 2025). Data were visually inspected before and after processing for quality assurance.

#### Anatomical Data Processing.

T1- and T2-weighted images were inhomogeneity corrected using *N4* (Tustison et al., 2010) and denoised using *ANTS* (Avants et al., 2011). The brain was then extracted using a combination of *BEaST* (Eskildsen et al., 2012) and brain-extracted and normalized reference brains from *IXI* (BIAC, 2022). Extracted T1 images were normalized to a variant of the 1-mm T1-weighted MNI152 template that was modified to remove extracerebral tissue (non-linear 6th-generation symmetric average; Grabner et al., 2006). Normalization was performed using the diffeomorphic approach implemented in *SyN* (version 2.3.4) (Avants et al., 2011). T2-weighted images were rigidly co-registered with the corresponding T1 prior to normalization. The brain extraction mask from the T1 was then applied. Tissue priors were unwarped to native space using the inverse diffeomorphic transformation (Lorio et al., 2016). Brain-extracted T1 and T2 images were segmented using native-space priors generated in *FAST* (version 6.0.4) for use in T1-EPI co-registration (Jenkinson et al., 2012).

#### Fieldmap Data Processing.

SE images and *topup* were used to create fieldmaps. Fieldmaps were converted to radians, median-filtered, and smoothed (2-mm). The average of the distortion-corrected SE images was inhomogeneity corrected using *N4* and masked to remove extracerebral voxels using *3dSkullStrip* (version 19.1.00). The resulting mask was minimally eroded to further exclude extracerebral voxels.

#### Functional Data Processing.

EPI files were de-spiked using *3dDespike*, slice-time corrected to the TR-center using *3dTshift*, and motion-corrected to the first volume and inhomogeneity corrected using *ANTS* (12-parameter affine). Transformations were saved in ITK-compatible format for subsequent processing (McCormick et al., 2014). The first volume was extracted for EPI-T1 co-registration. The reference EPI volume was simultaneously co-registered with the corresponding T1-weighted image in native space and corrected for geometric distortions using boundary-based registration (Jenkinson et al., 2012). This step incorporated the previously created fieldmap, undistorted SE, T1, white matter (WM) image, and masks. The spatial transformations necessary to transform each EPI volume from native space to the reference EPI, from the reference EPI to the T1, and from the T1 to the template were concatenated and applied to the processed EPI data in a single step to minimize incidental spatial blurring. Normalized EPI data were resampled (2 mm^3^) using fifth-order b-splines. Voxelwise analyses employed data that were spatially smoothed (4-mm) using *3DblurInMask*. To minimize signal mixing, smoothing was confined to the gray-matter compartment, defined using a variant of the Harvard-Oxford cortical and subcortical atlases that was expanded to include the bed nucleus of the stria terminalis (BST) and periaqueductal gray (PAG) (Desikan et al., 2006; Edlow et al., 2012; Frazier et al., 2005; Makris et al., 2006; Theiss et al., 2017). Focal analyses of the extended amygdala (EA) leveraged spatially unsmoothed data and anatomically defined regions of interest (ROIs; see below), consistent with prior work by our group (Cornwell et al., 2025).

### Skin Conductance Modeling

Robust general linear models (GLMs) were used to separate electrodermal signals associated with threat anticipation from those evoked by other aspects of the task (e.g., reinforcer presentation). Modeling was performed separately for each participant and scan using *robustfit*. Subject-specific SCRFs were convolved with rectangular regressors time-locked to the presentation of the reinforcers (separately for each trial type), visual masks, and rating prompts. The first-level residuals were then averaged separately for each participant and condition, enabling us to quantify skin conductance level (SCL) during the anticipation (‘countdown’) epochs.

### fMRI Data Modeling

#### First-Level Modeling.

For each participant, first-level modeling was performed using GLMs implemented in *SPM12* (version 7771), with the default autoregressive model and the temporal band-pass filter set to the hemodynamic response function (HRF) and 128 s (Wellcome Centre for Human Neuroimaging, 2022). Regressors were convolved with a canonical HRF and its temporal derivative. For the threat-anticipation paradigm, hemodynamic activity was modeled using variable-duration rectangular (‘boxcar’) regressors that spanned the entirety of the anticipation (‘countdown’) epochs of the Uncertain-Threat, Certain-Threat, and Uncertain-Safety trials. To maximize design efficiency, Certain-Safety anticipation served as the reference condition and contributed to the implicit baseline estimate. Epochs corresponding to the presentation of the four types of reinforcers, white-noise visual masks, and rating prompts were simultaneously modeled using the same approach. EPI volumes acquired before the first trial and following the final trial were unmodeled and contributed to the baseline estimate. Consistent with prior work (Cornwell et al., 2025), nuisance variates included volume-to-volume displacement and first derivative, 6 motion parameters and first derivatives, cerebrospinal fluid (CSF) signal, instantaneous pulse and respiration rates, and nuisance signals (e.g., brain edge, CSF edge, global motion, WM, and extracerebral soft tissue) (Anderson et al., 2011; Pruim et al., 2015). Volumes with excessive volume-to-volume displacement (>0.75 mm) and those during and immediately following reinforcer delivery were censored.

#### Anatomical ROIs.

Ce and BST activation was quantified using well-established, anatomically defined regions-of-interest (ROIs) and spatially unsmoothed data (Theiss et al., 2017; Tillman et al., 2018). The BST ROI mostly encompasses the supra-commissural BST, given the difficulty of reliably discriminating the sub-commissural BST border in standard anatomical images (Kruger et al., 2015; Walter et al., 1991). Bilateral ROIs were decimated to the 2-mm resolution of the fMRI data. ROI analyses used standardized regression coefficients extracted and averaged for each combination of task contrast (e.g., Uncertain-Threat anticipation vs. Uncertain-Safety anticipation), region, and participant. Anatomical ROIs enable statistically unbiased tests of regional sensitivity to specific experimental manipulations (i.e., Region × Condition effects), including potential single and double dissociations (e.g., BST: Uncertain > Certain Threat; Ce: Uncertain < Certain Threat) (Fox et al., 2018).

### Analytic Strategy

#### Overview.

Except where noted otherwise, analyses were performed using *SPM12* (version 7771) and *SPSS* (version 27.0.1.0) (Wellcome Centre for Human Neuroimaging, 2022). Diagnostic procedures and data visualizations were used to confirm that test assumptions were satisfied (Tukey, 1977). Standardized frequentist (Cohen’s *d*) effect sizes were interpreted using established benchmarks (Cohen, 1988; Cohen, 1994; Schimmack, 2019), ranging from *large* (*d*=0.80), to *medium* (*d*=0.50), to *small* (*d*=0.20), to *nil* (*d*≤0.10). Some figures were created using created using *ggplot2* (version 3.3.6) (Wickham, 2016) and *MRIcron* (Rorden, 2019). Clusters and local maxima were labeled using the Harvard–Oxford atlas (Desikan et al., 2006; Frazier et al., 2005; Makris et al., 2006), supplemented by other resources (ten Donkelaar et al., 2018).

*JASP* (version 0.16.4.0) was used to compute Bayesian effect sizes for select analyses (Love et al., 2019; van Doorn et al., 2021). Here, Bayes Factor (BF_10_) quantifies the relative performance of the null hypothesis (*H*_0_; e.g., the absence of a credible mean difference) and the alternative hypothesis (*H*_1_; e.g., the presence of a credible mean difference), on a 0 to ∞ scale. A key advantage of BF is that it can be used to quantify the relative strength of the evidence for *H*_0_ (test the null), unlike conventional null hypothesis significance tests (Bo et al., 2024; E.-J. Wagenmakers et al., 2018). It also does not require the data analyst to arbitrarily decide what constitutes a ‘statistically indistinguishable’ difference, in contrast to traditional equivalence tests (Hur et al., 2020). The Bayesian approach provides readily interpretable, principled effect-size benchmarks (van Doorn et al., 2021). Values >1 were interpreted as evidence of mean differences in activation across conditions, ranging from *strong* (*BF*_*10*_>10), to *moderate* (*BF*_*10*_=3–10), to *weak* (*BF*_*10*_=1–3). Values <1 were interpreted as evidence of statistical equivalence (i.e., support for the null hypothesis), ranging from *strong* (*BF*_*10*_<0.10), to *moderate* (*BF*_*10*_=0.10–0.33), to *weak* (*BF*_*10*_=0.33–1). The reciprocal of *BF*_*10*_ represents the relative likelihood of the null hypothesis (e.g., *BF*_*10*_=0.10, *H*_0_ is 10 times more likely than *H*_1_). Bayesian effects were computed using a noninformative zero-centered Cauchy distribution (*ω*=1/√2), the default setting in *JASP* and the field standard for two-sided tests (Gronau et al., 2020; Schmalz et al., 2023; Schönbrodt et al., 2017; van Doorn et al., 2021; E.-J. Wagenmakers et al., 2018).

#### Ratings and Psychophysiology.

We used repeated-measures general linear models (GLMs) to confirm that the threat-anticipation paradigm amplified subjective symptoms of distress (in-scanner fear/anxiety ratings) and objective signs of arousal (SCL). Interactions were probed using focal contrasts. Sensitivity analyses confirmed that none of the conclusions materially changed when controlling for potential nuisance variation in mean-centered study, age, and assigned sex (for additional details, see the study OSF collection).

#### Whole-Brain Voxelwise Tests.

Spatially smoothed (4-mm) data and whole-brain voxelwise (‘second-level’) repeated-measures GLMs (‘random effects’) were used to compare each threat-anticipation condition to its corresponding control condition (e.g., Uncertain-Threat vs. Uncertain-Safety anticipation), while accounting for potential nuisance variation in mean-centered study, age, and assigned sex. Significance was assessed using *p*<0.05 (whole-brain familywise error [FWE] corrected). A minimum conjunction test (logical ‘AND’) was used to identify the subset of voxels significantly sensitive to both Certain *and* Uncertain Threat anticipation (Nichols et al., 2005). We also directly examined potential differences in anticipatory activity between the two threat conditions (Certain Threat vs. Uncertain Threat). We did not examine hemodynamic responses to reinforcer presentation given the possibility of artifact.

#### Anatomical ROIs.

As a precursor to hypothesis testing, a series of one-sample Student’s *t*-tests was used to confirm that the EA (BST/Ce) ROIs—which leveraged spatially unsmoothed data—showed nominally significant recruitment during Certain and Uncertain Threat anticipation relative to their respective control conditions (*p*<0.05, uncorrected). For hypothesis testing, we used a standard 2 (*Region*: Ce, BST) × 2 (*Threat-Certainty*: Certain, Uncertain) repeated-measures GLM to test potential regional differences in activation during the anticipation of temporally Certain Threat (relative to Certain Safety) versus Uncertain Threat (relative to Uncertain Safety). These analyses leveraged spatially unsmoothed data to maximize anatomical resolution and inferential clarity. Interactions were probed using focal contrasts. Sensitivity analyses confirmed that none of the conclusions materially changed when controlling for potential nuisance variation in mean-centered study, age, and assigned sex (for additional details, see the study OSF collection). A sign test (*Z*_*Sign*_) was used to nonparametrically test the proportion of participants showing double dissociations.

## RESULTS

### Threat anticipation amplifies subjective distress and objective arousal

**Figure 2.**
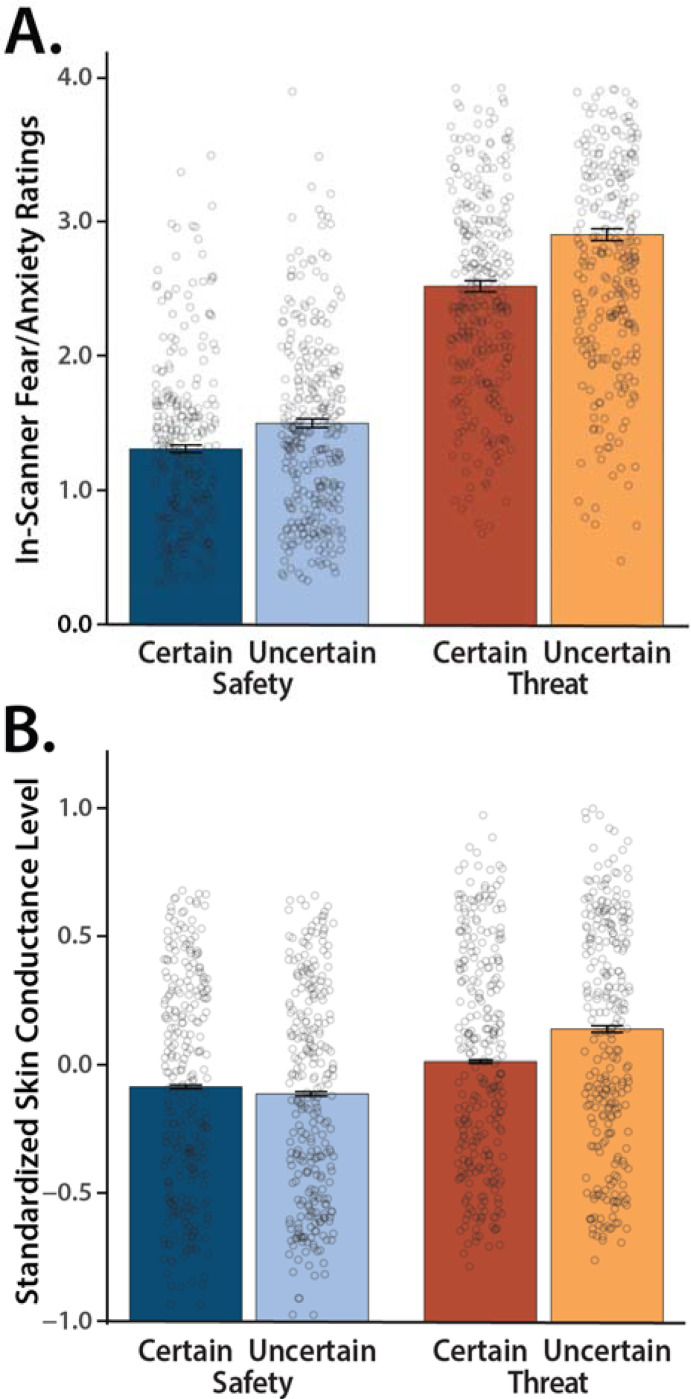
The Maryland Threat Countdown paradigm is a valid probe of human fear and anxiety. ***(A) Anticipated threat increases subjective symptoms of distress.*** Conscious feelings of fear and anxiety were increased during the anticipation of threat compared to safety, and this was particularly evident for temporally uncertain threat (*p*<0.001). ***(B) Anticipated threat increases objective signs of arousal.*** A similar pattern was evident for SCL (p<0.001). Bars depict means, whiskers depict standard errors, and open rings depict individual participants.

We used repeated-measures general linear models (GLMs) to confirm that the threat-anticipation paradigm had the intended impact on anticipatory distress (in-scanner ratings) and arousal (skin conductance level, SCL). Eight participants were excluded from SCL analyses due to insufficient usable data (*n*=287). As shown in Figure 2A, subjective feelings of fear and anxiety were significantly elevated during the anticipation of threat compared to safety, and distress was particularly pronounced when the timing of threat encounters was uncertain (*Valence*: *F*(1,294)=965.74, *p*<0.001, *d*=1.81, *BF*_*10*_=1.42×10^91^; *Certainty*: *F*(1,294)=231.95, *p*<0.001, *d*=0.89, *BF*_*10*_=3.91×1035; *Valence* × *Certainty*: *F*(1,294)=25.58, *p*<0.001, *d*=0.29, *BF*_*10*_=12,327.09; *Threat, Uncertain vs. Certain*: *F*(1,294)=154.04, *p*<0.001, *d*=0.72, *BF*_*10*_=3.14×10^25^; *Safety, Uncertain vs. Certain*: *F*(1,218)=77.63, *p*<0.001, *d*=0.38, *BF*_*10*_=4.42×10^13^).

As shown in Figure 2B, the same general pattern was evident for SCL, an objective psychophysiological index of anticipatory arousal (*Valence*: *F*(1,286)=165.76, *p*<0.001, *d*=0.76, *BF*_*10*_=9.61×10^26^; *Certainty*: *F*(1,286)=80.21, *p*<0.001, *d*=0.53, *BF*_*10*_=1.09×10^14^; *Valence × Certainty*: *F*(1,286)=129.87, *p*<0.001, *d*=0.67, *BF*_*10*_=7.53×10^21^; *Threat, Uncertain vs. Certain*: *F*(1,286)=120.97, p<0.001, *d*=0.65, *BF*_*10*_=3.49×10^20^; *Safety*, *Uncertain vs. Certain*: *F*(1,286)=43.61, *p*<0.001, *d*=0.39, *BF*_*10*_=3.57×10^7^). Taken together, these converging observations confirm the validity of the MTC paradigm as an experimental probe of human fear and anxiety, consistent with work in smaller samples (Hur et al., 2020; Kim et al., 2023).

### Uncertain-threat anticipation recruits a distributed cortico-subcortical network

**Figure 3.**
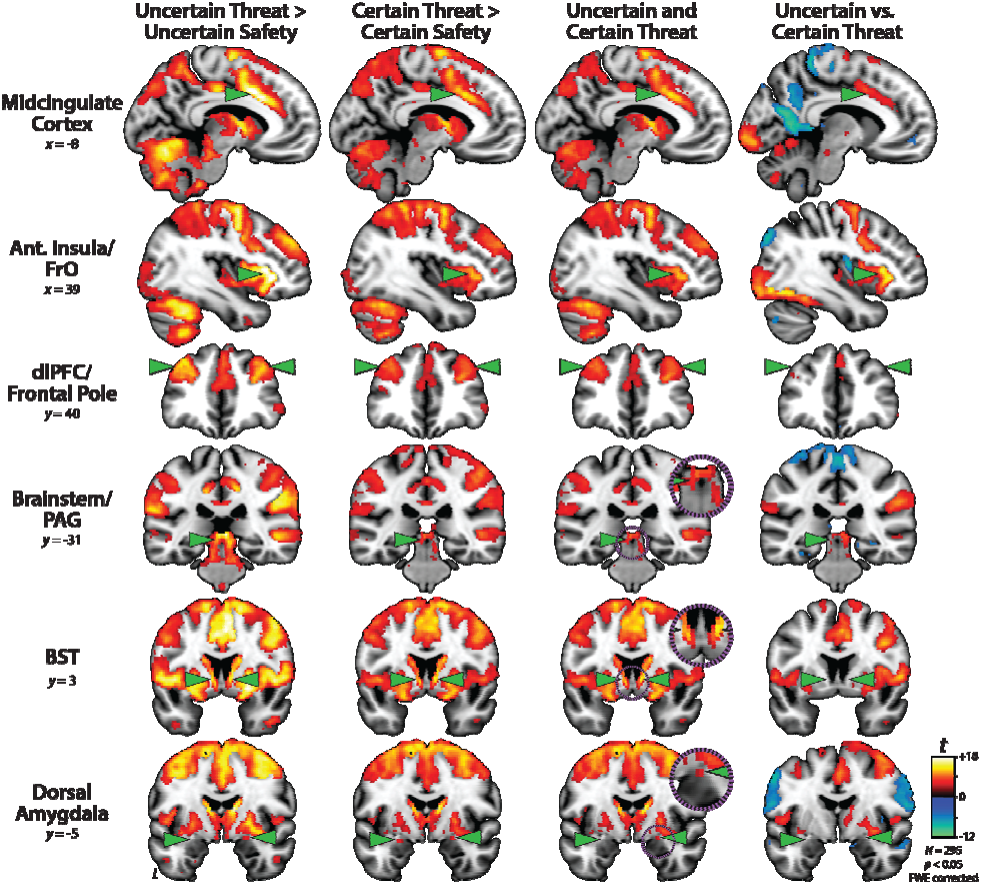
Uncertain- and certain-threat anticipation recruit a common cortico-subcortical network. Key regions (*green arrowheads*) show significantly increased activation during the anticipation of both uncertain threat (*first column*) and certain threat (*second column*), relative to their respective control conditions (*p*<0.05, whole-brain FWE corrected). *Third column* depicts the voxelwise conjunction (logical ‘AND’) of the two thresholded contrasts. Co-localization is evident throughout the network, including the BST and dorsal amygdala (Ce). *Fourth column* shows the direct contrast of the two threat-anticipation conditions. The MCC, AI/FrO, and to a lesser extent dlPFC/FP, show significantly greater activation during the anticipation of uncertain threat, whereas the BST, dorsal amygdala (Ce), and PAG show negligible discrimination of the two conditions. The dlPFC/FP mean difference was more evident at more rostral planes. For additional details, see Supplementary Tables S1-S6. *Purple insets* depict magnified views of overlap in the PAG, BST, and Ce. Abbreviations—Ant., Anterior; BST, Bed Nucleus of the Stria Terminalis; dlPFC, Dorsolateral Prefrontal Cortex; FrO, Frontal Operculum; FWE, Familywise Error; PAG, Periaqueductal Gray.

We used a whole-brain voxelwise GLM to identify regions recruited during the anticipation of temporally uncertain threat, relative to uncertain safety (*p*<0.05, whole-brain FWE corrected). As shown in the first column of Figure 3, this revealed a widely distributed network of cortical and subcortical regions previously implicated in the expression and regulation of human fear and anxiety (Bo et al., 2024; Chavanne & Robinson, 2021; Grogans et al., 2024; Hur et al., 2022; Radua et al., in press; Shackman & Fox, 2021), including the midcingulate cortex (MCC); anterior insula (AI) extending into the frontal operculum (FrO); dorsolateral prefrontal cortex (dlPFC) extending to the frontal pole (FP); brainstem encompassing the periaqueductal grey (PAG); basal forebrain, in the region of the BST; and dorsal amygdala, in the region of the Ce (Supplementary Table S1).

While not the focus of our study, exploratory analyses indicated that uncertain-threat anticipation was associated with reduced activation (‘de-activation’) in a set of midline regions that encompassed key nodes of the default mode network (e.g., frontal pole, rostral and straight gyri, and precuneus) as well as the pre- and post-central gyri, posterior insula, parahippocampal gyrus, and hippocampus (Supplementary Table S2). At a more liberal threshold (FDR *q*<0.05), the same pattern was evident in ventromedial regions of the amygdala (e.g., basal and cortical nuclei and amygdalohippocampal transition area), consistent with prior neuroimaging studies of anticipated threat and with the known functional heterogeneity of this complex structure (Cornwell et al., 2025; Fox & Shackman, 2024; Murty et al., 2022; Murty et al., 2023).

### Certain-threat anticipation recruits a broadly similar network

We used a parallel approach to identify regions recruited during the anticipation of temporally certain threat, relative to certain safety (*p*<0.05, whole-brain FWE corrected). As shown in the second column of Figure 3, the results strongly overlapped those evident for Uncertain Threat (Supplementary Tables S3-S4). In fact, a minimum-conjunction analysis of the two contrasts (Logical ‘AND;’ Nichols et al., 2005) revealed voxelwise colocalization in every key region, including the BST and dorsal amygdala in the region of the Ce (third column of Figure 3). Taken together, these results suggest that this distributed cortico-subcortical system is sensitive to multiple kinds of anticipated threat, including those that are certain and uncertain in their timing.

### Fronto-cortical regions discriminate uncertain from certain threat, subcortical regions do not

To determine whether regions recruited during threat anticipation are sensitive to temporal uncertainty, we directly compared the uncertain and certain threat conditions (*p*<0.05, whole-brain FWE corrected). As shown in the fourth column of Figure 3, fronto-cortical regions—including MCC, AI/Fro, and dlPFC/FP—while engaged by both kinds of threat, showed a preference for temporally uncertain threat, consistent with prior work (Supplementary Tables S5-S6) (Hur et al., 2020). In contrast, the BST, dorsal amygdala (Ce), and PAG showed negligible differences.

### The BST and Ce show statistically indistinguishable responses to certain- and uncertain-threat anticipation

**Figure 4.**
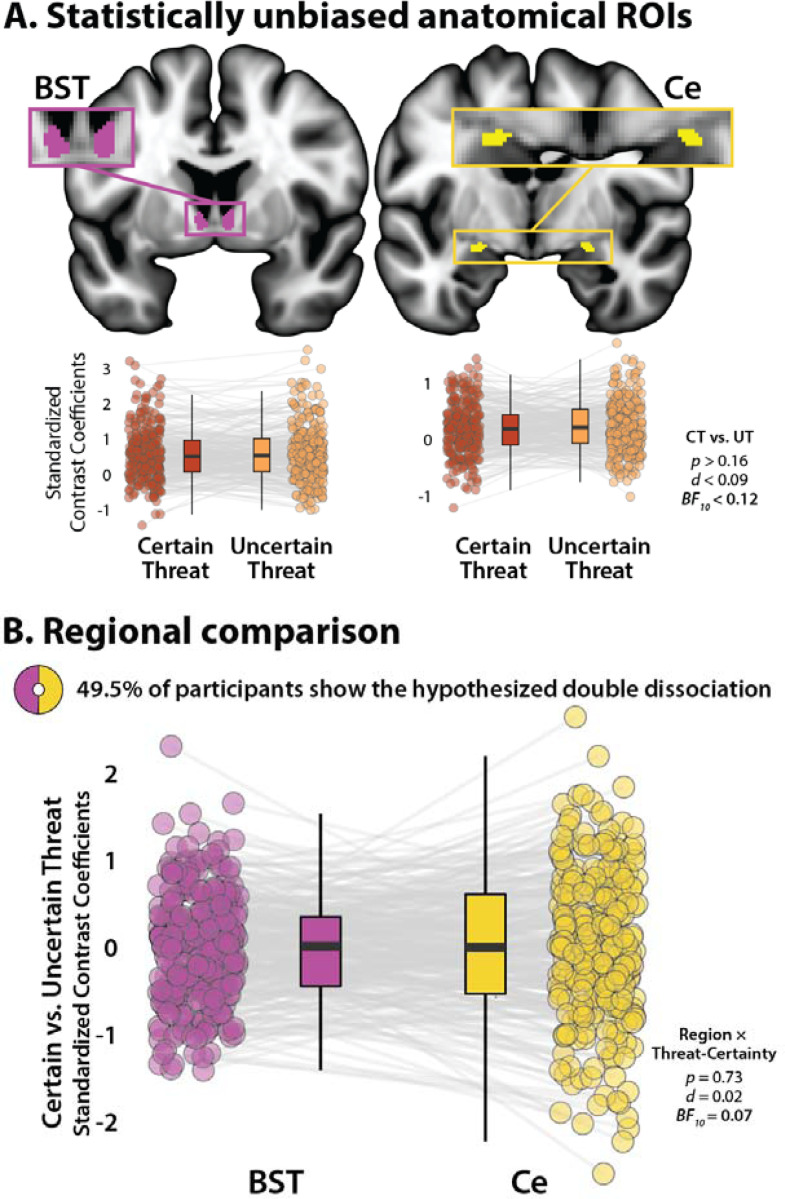
The human BST and Ce show statistically indistinguishable responses during certain- and uncertain-threat anticipation. **(A) *Anatomical ROIs.*** Probabilistic anatomical ROIs provided statistically unbiased estimates of BST and Ce activation during certain- and uncertain-threat anticipation. Leveraging spatially unsmoothed data, regression coefficients were extracted and averaged across voxels for each combination of ROI, task contrast, and participant. Box plots underscore negligible activation differences during the anticipation of certain-versus-uncertain threat in both the BST *(left)* and the Ce *(right),* contrary to double-dissociation models (*p*>0.16, *d*<0.09, *BF*_*10*_<0.12], Note: The *y*-axis scale differs across ROIs. **(B) *Regional comparison.*** A standard repeated-measures GLM was used to directly assess potential regional differences in reactivity to certain-versus-uncertain threat. Contrary to the double-dissociation model, the Region x Threat-Certainty interaction was not significant. Boxplot depicts the interaction as a 1-*df* contrast, that is, the ‘difference of differences’. Participants were just as likely as not (49.5% vs. 50.5%) to show the hypothesized double dissociation, *Z*_*Sign*_=0.12, *p*=0.91. Boxplots depict the median *(horizontal lines),* interquartile range *(boxes),* and individual participants *(dots)* for each contrast. Whiskers indicate 1.5× the interquartile range. Gray lines depict the sign and magnitude of intra-individual mean differences. Inset ring plot depicts the percentage of participants showing the hypothesized dissociation of regional reactivity to threat (BST: Certain < Uncertain Threat; Ce: Certain > Uncertain Threat). Abbreviations—BF, Bayes Factor; BST, bed nucleus of the stria terminalis; Ce, central nucleus of the amygdala; CT, certain-threat anticipation; *d,* Cohen’s *d.* fMRI, functional magnetic resonance imaging; FWE, familywise error; GLM, general linear model; ROI, region-of-interest; UT, uncertain-threat anticipation.

Because voxelwise analyses do not permit inferences about regional differences in activation, we used anatomical regions of interest (ROIs) and spatially unsmoothed data to rigorously compare the BST and Ce (Figure 4A). As a precursor to hypothesis testing, we used one-sample t-tests to confirm that the BST and Ce ROIs show significant activation during certain- and uncertain-threat anticipation relative to their respective control conditions (*t*(294)>6.49, *p*<0.001, *d*>0.37, *BF*_*10*_>2.02 × 10^7^). Next, we used a standard 2 (*Region*: BST, Ce) × 2 (*Threat-Certainty*: Certain, Uncertain) repeated-measures GLM to probe potential regional differences in threat sensitivity. Here again the BST and Ce proved statistically indistinguishable (Figure 4A, Supplementary Figure S2A-B). The critical Region × Threat-Certainty contrast was not significant (*F*(1,294)=0.12, *p*=0.73, *d*=0.02, *BF*_*10*_=0.07). In fact, participants were just as likely as not (49.5% vs. 50.5%; *H_0_*=50.0%) to show the hypothesized double-dissociation pattern (*Z*_*Sign*_=0.12, *p*=0.91; Figures 4B, Supplementary Figure S2C). Focal contrasts indicated that neither the BST nor the Ce credibly discriminated certain-from uncertain-threat anticipation (*BST*: *t*(294)=0.59, *p*=0.56, *d*=0.03, *BF*_*10*_=0.11; *Ce*: *t*(294)=1.38, *p*=0.17, *d*=0.08, *BF*_*10*_=0.03; Figure 4A), consistent with the more conservatively thresholded voxelwise results (Figure 3). The GLM did, however, reveal a main effect of region, reflecting generally greater BST reactivity to both kinds of threat anticipation (*F*(1,294)=95.36, *p*<0.001, *d*=0.57, *BF*_*10*_=3.95 × 10^16^). The main effect of Threat-Certainty was not significant (*p*=0.22, *d*=0.07, *BF*_*10*_=0.14). None of the conclusions materially changed when mean-centered study, age, and biological sex were included as nuisance variates. In short, when viewed through the lens of hemodynamics and the MTC paradigm, the BST and Ce show statistically indistinguishable responses to certain- and uncertain-threat anticipation.

## DISCUSSION

In the realm of human neuroimaging research, the present results provide some of the strongest evidence to date that the functional architecture of the EA does not conform to popular double-dissociation models (Supplementary Figure S1 and Note 1; Avery et al., 2016; Klumpers et al., 2017; LeDoux & Pine, 2016; NIMH, 2011; Somerville et al., 2013). The Ce and BST are both engaged during periods of threat anticipation, and the degree of engagement is independent of the temporal certainty of threat encounters. In a head-to-head comparison, the two regions showed statistically indistinguishable selectivity for the two kinds of threat (*d*=0.02), with strong Bayesian evidence of regional equivalence (*BF*_*10*_=0.07; *H_0_* is 14.3 times more likely than *H_1_*). These functional similarities are consistent with other evidence. The Ce and BST are characterized by similar patterns of anatomical connectivity, cellular composition, neurochemistry, and gene expression (Fox, Oler, Tromp, et al., 2015). Both are poised to trigger behavioral, psychophysiological, and neuroendocrine responses to threat via dense projections to downstream effector regions (Davis & Whalen, 2001; Fox, Oler, Tromp, et al., 2015). Activity in both regions has been shown to co-vary with individual differences in trait anxiety in large-scale studies of nonhuman primates (*n*=592) (Fox, Oler, Shackman, et al., 2015). Among humans, the Ce and BST are recruited by a broad spectrum of threatening and aversive stimuli (Fox & Shackman, 2019; Hudson et al., 2020; Hur et al., 2020; Radua et al., *in press*; Shackman & Fox, 2021; Sjouwerman et al., 2020) and both regions show hyper-reactivity to emotional tasks in individuals with anxiety disorders (Chavanne & Robinson, 2021; Shackman & Fox, 2021). Mechanistic work in rodents demonstrates that microcircuits within and between the Ce and BST are critical governors of defensive responses to both certain and uncertain threats (Chen et al., 2022; Fox & Shackman, 2019; Holley & Fox, 2022; Lange et al., 2017; Moscarello & Penzo, 2022; Pomrenze, Giovanetti, et al., 2019; Pomrenze, Tovar-Diaz, et al., 2019; Ren et al., 2022; Ressler et al., 2020; Zhu et al., 2024). In fact, work in mice using a variant of the MTC shows that projections from the Ce to the BST are necessary for mounting defensive responses to temporally uncertain threat (Lange et al., 2017), dovetailing with the present results. Although our understanding remains far from complete, this body of work underscores the need to reformulate models that posit a strict functional segregation of certain and uncertain threat processing in the EA.

These observations do not mean that the Ce and BST are functionally identical or interchangeable (Ressler et al., 2020; Tseng et al., 2023; Wang et al., 2024). In fact, our results indicate that the BST is more strongly recruited by both kinds of anticipated threat. Work in monkeys demonstrates that BST activity is more closely related to heritable variation (‘nature’) in trait anxiety, whereas Ce activity is more closely related to the variation in trait anxiety that is explained by differences in early-life experience (‘nurture’) (Fox, Oler, Shackman, et al., 2015). The BST also appears to be more closely involved in organizing persistent signs of fear and anxiety following threat encounters (i.e., mood ‘spillover;’ Shackman et al., 2017). Among humans, individual differences in neuroticism/negative emotionality, a prominent risk factor for anxiety disorders and depression, is selectively associated with heightened BST reactivity to uncertain-threat anticipation, an association that remains evident when controlling for Ce reactivity (Grogans et al., 2024). Clarifying the nature of these regional differences is an important avenue for future research. This endeavor is likely to benefit from the use of pharmacological challenges (e.g., acute benzodiazepine; Grillon & Ernst, 2020; Miles et al., 2011) and computational modeling (Holley & Fox, 2022), two approaches that would facilitate the development of well-coordinated cross-species models of fear and anxiety. Computational modeling, in particular, has the potential to address fundamental questions about the function of threat-sensitive brain regions, clarify inconsistencies across paradigms, and foster a common mathematical framework (‘*lingua franca’*) for unifying research across investigators, readouts, and species (Drzewiecki & Fox, 2024).

The processing of uncertain and certain anticipated threats is hardly confined to the EA. In fact, exploratory whole-brain voxelwise analyses revealed a distributed network that encompasses both frontocortical (MCC, AI/FrO, and dlPFC/FP) and other subcortical regions (PAG) regions (Figure 3). And while this network is sensitive to both kinds of threat, with co-localization evident in every key region, direct comparison of the two threat conditions revealed greater frontocortical activation during uncertain-threat anticipation. We previously hypothesized that this could reflect differences in either cognitive load or the intensity of distress and arousal across conditions (Hur et al., 2020). On certain-threat trials, the descending integer stream (‘countdown’) provides an overt index of momentary changes in threat imminence. On uncertain-threat trials, this cognitive scaffolding is absent, encouraging reliance on the kinds of sustained, endogenous representations that are the hallmark of frontocortical regions (Badre & Nee, 2018). A second notable difference between the two threat conditions is the greater intensity of distress and arousal elicited by uncertain threat (Figure 2), potentially reflecting differences in the cumulative hazard rate (i.e., the mathematical probability of encountering threat, given that it has not yet occurred) across the two threat conditions (Holley et al., 2024). From this perspective, increased frontocortical activation could reflect either heightened fear/anxiety or stronger recruitment of compensatory processes aimed at downregulating negative affect. On the one hand, there is ample evidence that frontocortical regions, including the MCC and AI/FrO, are recruited by a wide variety of aversive challenges, consistent with a role in *generating* negative affect (Bo et al., 2024; Chavanne & Robinson, 2021; Radua et al., in press; Xu et al., 2020). Yet frontocortical regions also play a role in *regulating* distress (Bo et al., 2024). In laboratory studies of instructed emotion regulation, MCC and dlPFC/FP activation scales with the degree of regulatory success (Bo et al., 2024; Urry et al., 2009), raising the possibility that frontocortical activation during aversive laboratory challenges partially reflects spontaneous efforts to downregulate or inhibit distress (‘implicit’ regulation; Shackman & Lapate, 2018). Consistent with this hypothesis, we recently showed that heightened MCC and FrO reactivity to the MTC paradigm is associated with dampened emotional reactivity to real-world stressors, indexed using ecological momentary assessment (Hur et al., 2022), an observation that is consistent with evidence that loss of MCC function is associated with increased (‘dysregulated’) emotional reactivity to painful stimuli in humans and amplified defensive responses to threat in monkeys (Davis et al., 1994; Greenspan et al., 2008; Rahman et al., 2021).

Clearly, several challenges remain for future research. First, it will be important to determine whether our conclusions generalize to more demographically representative samples, other types of experimental threat (e.g., social), other kinds of uncertainty (e.g., probability, risk, ambiguity), and more naturalistic paradigms that span longer and more ecologically valid periods of threat anticipation (Mobbs et al., 2021). It merits comment that the absence of reward trials precludes strong claims about valence.

Second, the Ce and BST are complex and can be subdivided into multiple subdivisions, each containing intermingled cell types with distinct, even opposing functional roles (e.g., anxiogenic vs. anxiolytic) (Fox & Shackman, 2019, 2024; Holley & Fox, 2022; Moscarello & Penzo, 2022). Animal models will be critical for generating testable hypotheses about the most relevant molecules, cell types, and microcircuits (Fox & Shackman, 2019, 2024; Kamboj et al., 2024). Third, fear and anxiety reflect the coordinated interactions of widely distributed neural networks (Mineka et al., 2020; Monroe & Harkness, 2022; Tozzi et al., 2024; Zhang et al., 2023). Moving forward, it will be important to clarify the relevance of functional connectivity within and beyond the EA.

Anxiety disorders impose a staggering burden on global health, afflicting ~360 million individuals annually (GBD, 2024). Existing treatments were developed decades ago and have limited efficacy, durability, and tolerability (Batelaan et al., 2017; Cuijpers et al., 2024; Singewald et al., 2023; Strawn et al., 2021). Rising to this challenge requires the development of more accurate models of the neural systems governing fear and anxiety in health and disease. Leveraging a well-powered mega-analytic sample, translationally relevant fMRI paradigm, and best-practices analytic approach, the present results demonstrate that the EA systems recruited by certain and uncertain threat are not categorically different, with clear evidence of functional colocalization—*not* segregation—in the Ce and BST. These observations provide an empirically grounded framework for conceptualizing fear and anxiety, for understanding the functional neuroanatomy of threat processing in humans, and for accelerating the development of improved biological interventions for the suffering caused by pathological fear and anxiety.

## Supplementary Material

Supplement 1
