# Supplementary material for: Fear, anxiety, and the extended amygdala—Absence of evidence for strict functional segregation
Source: bioRxiv. 2025 Sep 4:2025.08.29.673083. Preprint. [Version 1] doi: 10.1101/2025.08.29.673083 (PMC12424656; doi:10.1101/2025.08.29.673083)
Supplement: Supplementary file 1 [file media-1.zip › didier_shackman_PaxSaxMega_SCAN_Suppl_082525b.docx]

**Supplementary Materials**

Paige R. Didier^1^

Shannon E. Grogans^1^

Claire M. Kaplan^4^

Hyung Cho Kim^2,3^

Samiha Islam^5^

Allegra S. Anderson^6^

Rachael M. Tillman^7^

Manuel Kuhn^8^

Juyoen Hur^9^	

Andrew S. Fox^10,11^

Kathryn A. DeYoung^1^

Jason F. Smith^1^

Alexander J. Shackman^1,2,3^

Department of ^1^Psychology, ^2^Neuroscience and Cognitive Science Program, and ^3^Maryland Neuroimaging Center, University of Maryland, College Park, MD 20742 USA. ^4^Department of Psychiatry and Behavioral Sciences, School of Medicine, Johns Hopkins University, Baltimore, MD 21205 USA. ^5^Department of Psychology, University of Pennsylvania, Philadelphia, PA USA. ^6^Department of Psychiatry and Human Behavior, Brown University, Providence, RI 02912 USA. ^7^McGill Neuropsychology, Bethesda, MD 20814 USA. ^8^Center for Depression, Anxiety and Stress Research, McLean Hospital, Harvard Medical School, Belmont, MA 02478 USA. ^9^Department of Psychology, Yonsei University, Seoul 03722, Republic of Korea. ^10^Department of Psychology and ^11^California National Primate Research Center, University of California, Davis, CA 95616 USA

**Please address manuscript correspondence to** 

Alexander J. Shackman ([shackman@umd.edu](mailto:shackman@umd.edu))

**SUPPLEMENTARY NOTE 1.** ***Fear, anxiety, and the extended amygdala.***

In this section, we briefly highlight some prominent neuroscientific definitions of fear and anxiety and recent theoretical claims about the functional organization of the two major subdivisions of the extended amygdala, the central nucleus of the amygdala (Ce/CeA) and the bed nucleus of the stria terminalis (BST/BNST).  

By design, this brief survey is meant to be illustrative, *not* comprehensive. For a panel discussion of related conceptual issues, see Grogans and colleagues (Grogans et al., 2023). For an overview of popular assays for assessing fear and anxiety in different species, see Shackman and colleagues (Shackman et al., 2016). For an overview of concerns centered on the imprecision and inconsistency of fear-and-anxiety terminology see Perusini and Fanselow (Perusini & Fanselow, 2015) and Shackman and Fox (Shackman & Fox, 2016).   

Avery et al. *Neuropsychopharmacology* 2016 (Avery et al., 2016)

- *“The amygdala mediates short-term, phasic responses to immediate threats, whereas the BNST mediates sustained responses to contextual, diffuse, and unpredictable threats.”*

Daniel-Watanabe & Fletcher *Biological Psychiatry Global Open Science* 2022 (Daniel-Watanabe & Fletcher, 2022)

- *“Fear is related to the presence, or imminent presence, of the aversive stimulus, while anxiety is considered the more protracted state produced by a sustained expectation that the aversive event is likely to occur.”*

- *“Evidence that both amygdala and BNST are responsive to both predictable and unpredictable threats… calls into question this overall claim for a neural distinction between fear and anxiety.”*

- *“It is difficult to escape the conclusion that the current distinction between fear and anxiety is an unreliable one. While it has been useful in guiding research and clinical work, the inconsistencies suggest that there is a need to reexamine the distinction and consider the importance of other aspects of the experience of anxiety, such as uncertainty and avoidance.”*

  

Davis et al. *Neuropsychopharmacology* 2010 (Davis et al., 2010)

- *“Although the symptoms of fear and anxiety are very similar, they differ in terms of certain key dimensions….Fear is prompted by imminent and real danger, and galvanizes active defensive responses. In contrast, anxiety is often elicited by less specific and less predictable threats, or by those that are physically or psychologically more distant.”* 

- *“The BLA [basolateral amygdala] sends heavy projections to both the CeA and the BNST… The heaviest projections…are to the CeA*_*M*_ *[medial division of the Ce/CeA], which in turn projects to the hypothalamus and brainstem to mediate phasic fear responses…A long duration fear stimulus activates the BLA, which then rapidly activates the CeA*_*M*_ *to produce phasic fear through the release of glutamate acting on AMPA/kainate receptors on CeA*_*M*_ *neurons. Shortly thereafter, inputs to the CeA*_*L*_ *[lateral division of the Ce/CeA] then release CRF into the BNST to cause a long-lasting sustained fear reaction. Inhibitory feedback to the CeA*_*M*_ *from either the CeA*_*L*_ *or the BNST shuts down the CeA*_*M*_*, allowing a seamless transition from phasic to sustained fear.*

Domschke *Biological Psychiatry Global Open Science* 2022 (Domschke, 2022)

- *“Fear and anxiety seem to represent closely interrelated diagnostic constructs remaining to be further interrogated for their shared and unique molecular, neuronal, physiological, and behavioral substrates—quite in accordance with Martin Heidegger’s reconciling notion:* “Of course it still remains obscure how [anxiety] is connected ontologically with fear. Obviously these are kindred phenomena.”

Fox & Shackman *Neuroscience Letters* 2019 (Fox & Shackman, 2019)

- *“On balance, the brain imaging literature suggests that the Ce and BST, while certainly not interchangeable, are more alike than different.”*

- *“The central extended amygdala plays a crucial role in evaluating and responding to a broad spectrum of threat-related cues and contexts. While they are certainly not interchangeable, the Ce and the BST show similar patterns of connectivity, cellular composition, neurochemistry, and gene expression. Both are sensitive to uncertain or temporally remote threat; both co-vary with threat-elicited changes in behavior, physiology, and experience; both show phasic responses to acute threat; and both show heightened activity during sustained exposure to diffusely threatening contexts. Work in rodents indicates that both regions play a critical role in organizing sustained defensive responses to a range of potentially threatening cues and contexts.”*

Grupe & Nitschke *Nature Reviews Neuroscience* 2013 (Grupe & Nitschke, 2013) 

- *“Fear and anxiety can be distinguished according to how much certainty one has regarding the likelihood, timing or nature of a future threat…Environmental cues indicating the unambiguous presence of an immediate threat give rise to intense 'fearful' defensive behaviours (that is, 'fight or flight'), whereas more diffuse, distal or unpredictable threat cues produce 'anxious' risk assessment behaviour…that is likely to persist until such uncertainty is resolved.”* 

- *“We define anxiety…as anticipatory affective, cognitive and behavioural changes in response to uncertainty about a potential future threat.”*

Gungor & Paré *Journal of Neuroscience* 2016 (Gungor & Paré, 2016) 

- *Early work stressed the differing involvement of the central amygdala (CeA) and bed nucleus of the stria terminalis (BNST) in the genesis of fear versus anxiety, respectively…This model became extremely influential and now guides a new wave of studies on the role of BNST in humans. Here, we consider evidence for and against this model…This analysis leads us to conclude that BNST's influence is not limited to the generation of anxiety-like responses to diffuse threats, but that it also shapes the impact of discrete threatening stimuli.”*

LeBow & Chen *Molecular Psychiatry* 2016 (Lebow & Chen, 2016)

- *“The amygdala is responsible for mediating specific cue-based fear responses, and thus controls the assessment of immediate or phasic fear.”* 

- *“Fear and anxiety can be divided into an immediate threat, for example, the presence of a predator, and pre- or post-encounter threats, for example, the apprehension of encountering a predator again. The BNST is hypothesized to mediate these longer-term responses to anxiety, where a challenge or recovery from a challenge to homeostasis occurs. In contrast, the CeA is thought to mediate shorter duration phasic fear responses, which occur in response to a current challenge to homeostasis.”*

LeDoux & Pine *American Journal of Psychiatry* 2016 (LeDoux & Pine, 2016)

- *“We propose…that the mental state term* fear *be used to describe feelings that occur when the source of harm, the threat, is either immediate or imminent, and* anxiety *be used to describe feelings that occur when the source of harm is uncertain or is distal in space or time.”*

- *“Just as findings demonstrating that the amygdala detects and controls behavioral and physiological responses to immediate threats have supported views of the amygdala as fear-circuit hub, other findings, about responses to uncertain threats, have led to a view of the circuitry of anxiety. Thus, in recent years, animal research has suggested the bed nucleus of the stria terminalis (BNST) is engaged when threats are uncertain…resulting in behavioral inhibition and risk assessment …The BNST has thus come to be for anxiety what the amygdala is for fear—a circuit hub out of which anxious feelings emerge.”*

Mobbs et al. *Trends in Cognitive Sciences* 2020 (Mobbs et al., 2020)

- *“Anxiety: a future-oriented emotional state associated with potential and uncertain threats.”*

- *“Fear: an emotion that is associated with a present and identifiable threat.”* 

Moscarello & Penzo *Nature Neuroscience* 2022 (Moscarello & Penzo, 2022)

- *In nature, animals display defensive behaviors that reflect the spatiotemporal distance of threats. Laboratory-based paradigms that elicit specific defensive responses in rodents have provided valuable insight into the brain mechanisms that mediate the construction of defensive modes with varying degrees of threat imminence….High- and low-imminence defensive modes…are mediated at the neural-circuit level within the CeA and its downstream targets.*

NIMH *Negative Valence Systems: Workshop Proceedings* 2011 (NIMH, 2011)

- *“Responses to acute threat (Fear): Activation of the brain’s defensive motivational system to promote behaviors that protect the organism from perceived danger. Normal fear involves a pattern of adaptive responses to conditioned or unconditioned threat stimuli (exteroceptive or interoceptive). Fear can involve internal representations and cognitive processing, and can be modulated by a variety of factors.”*

- *“Responses to potential harm (Anxiety): Activation of a brain system in which harm may potentially occur but is distant, ambiguous, or low/uncertain in probability, characterized by a pattern of responses such as enhanced risk assessment (vigilance). These responses to low imminence threats are qualitatively different than the high imminence threat behaviors that characterize fear.”*

- *“While there is considerable overlap in the efferent activity generated, the distinction between these two constructs has found support in both animal and human literatures. In animal models, acute threat is associated with efferent activity from the amygdala, whereas potential harm has been shown to be related to activation of the bed nucleus of the stria terminalis (BNST).”*

Tovote et al. *Nature Reviews Neuroscience* 2015 (Tovote et al., 2015)

- *“Whereas fear is evoked by discrete and acutely threaten**ing stimuli, anxiety can be operationalized as an emo**tional response to vague, potential threats**.”*

**SUPPLEMENTARY FIGURES AND CAPTIONS**

**Supplementary Figure S1.** Conceptual overview of competing hypotheses about the functional architecture of the extended amygdala (EA). **(A)** ***Double-dissociation model.*** Several influential models suggest that fear and anxiety arise from two strictly segregated, doubly dissociable neural systems, positing that the Ce is sensitive to certain (but not uncertain) threat and promotes signs and symptoms of fear; whereas the BST is sensitive to uncertain (but not certain) threat and promotes anxiety (see **Supplementary Note 1**, above). **(B)** ***Proposed model.*** We propose a competing hypothesis, positing that that the Ce and BST play a role in governing responses to both kinds of threat (Fox & Shackman, 2019; Shackman & Fox, 2016). Note: This figure is purely for heuristic purposes and does not depict actual data. Abbreviations—BST, bed nucleus of the stria terminalis; Ce, central nucleus of the amygdala. 

   

**Supplementary Figure S2.** ***The BST and Ce show statistically indistinguishable responses to threat.*** **(A)** ***BST.*** The raincloud plot depicts BST reactivity to Certain and Uncertain Threat. Ring plot indicates the percentage of participants showing greater BST activation during Certain versus Uncertain threat anticipation. **(B)** ***Ce.*** The raincloud plot depicts BST reactivity to Certain and Uncertain Threat. Ring plot indicates the percentage of participants showing greater Ce activation during Certain versus Uncertain threat anticipation. Raincloud plots depict the median (*horizontal lines*), interquartile range (*boxes*), individual participants (*dots*), and smoothed data distributions (*half violins*) for each contrast. Whiskers indicate 1.5× the interquartile range. Gray lines depict the sign and magnitude of intra-individual mean differences. Abbreviations—BF, Bayes Factor; BST, bed nucleus of the stria terminalis; Ce, central nucleus of the amygdala; *d*, Cohen’s *d*. **(C)** ***Testing regional differences.*** The raincloud plot depicts the Region × Condition interaction as a 1-*df* contrast (‘difference of differences’). Ring plot indicates the percentage of participants showing the RDoC-hypothesized dissociation of regional reactivity to threat (BST: Certain < Uncertain Threat; Ce: Certain > Uncertain Threat).

**SUPPLEMENTARY TABLES**

**Supplementary Table S1.** Descriptive statistics for clusters and local extrema showing greater activation during the anticipation of Uncertain Threat relative to Uncertain Safety (FWE *p*<0.05, whole-brain corrected, 4-mm smoothing kernel).

|  |  | **mm**^**3**^ | ***t*** | **x** | **y** | **z** |
| --- | --- | --- | --- | --- | --- | --- |
|  | **Cluster 1** | 516,112 |  |  |  |  |
|  | L Frontal Operculum Cortex |  | 21.66 | -40 | 14 | 2 |
|  | L Insular Cortex |  | 20.23 | -32 | 24 | 6 |
|  | L Cingulate Gyrus, anterior division |  | 19.08 | -6 | 10 | 40 |
|  | L Paracingulate Gyrus |  | 18.97 | -8 | 12 | 38 |
|  | L Temporal Occipital Fusiform Cortex |  | 18.48 | -28 | -60 | -18 |
|  | L Brain Stem |  | 16.62 | -2 | -28 | -2 |
|  | L Left Putamen |  | 16.13 | -22 | 8 | -4 |
|  | L Central Opercular Cortex |  | 15.88 | -52 | 4 | 2 |
|  | L Supramarginal Gyrus, anterior division |  | 15.38 | -64 | -32 | 22 |
|  | L Superior Frontal Gyrus |  | 15.32 | -14 | 2 | 70 |
|  | L Cingulate Gyrus, posterior division |  | 15.25 | -10 | -22 | 40 |
|  | L Supramarginal Gyrus, posterior division |  | 15.12 | -58 | -50 | 34 |
|  | L Precentral Gyrus |  | 15.11 | -40 | -4 | 48 |
|  | L Middle Frontal Gyrus |  | 14.70 | -40 | -2 | 58 |
|  | L Thalamus |  | 14.55 | -10 | -6 | 14 |
|  | L Juxtapositional Lobule Cortex |  | 14.53 | -8 | 2 | 52 |
|  | L Caudate |  | 14.39 | -10 | 0 | 10 |
|  | L Parietal Operculum Cortex |  | 12.56 | -62 | -26 | 18 |
|  | L Superior Parietal Lobule |  | 11.84 | -20 | -50 | 64 |
|  | L Precuneus Cortex |  | 11.64 | -10 | -48 | 52 |
|  | L Postcentral Gyrus |  | 11.22 | -58 | -22 | 24 |
|  | L Inferior Frontal Gyrus, pars opercularis |  | 11.12 | -58 | 10 | 18 |
|  | L Middle Temporal Gyrus, temporooccipital part |  | 10.57 | -46 | -60 | 10 |
|  | L Lateral Occipital Cortex, inferior division |  | 10.04 | -54 | -66 | 10 |
|  | L Lingual Gyrus |  | 9.94 | -12 | -86 | -12 |
|  | L Occipital Pole |  | 9.26 | -20 | -96 | 12 |
|  | L Lateral Occipital Cortex, superior division |  | 9.03 | -32 | -60 | 58 |
|  | L Angular Gyrus |  | 8.97 | -48 | -54 | 52 |
|  | L Pallidum |  | 7.60 | -26 | -14 | -2 |
|  | L Planum Polare |  | 7.35 | -42 | -2 | -14 |
|  | L Temporal Fusiform Cortex, posterior division |  | 6.50 | -44 | -40 | -22 |
|  | L Temporal Pole |  | 5.87 | -46 | 18 | -22 |
|  | R Paracingulate Gyrus |  | 20.98 | 8 | 20 | 36 |
|  | R Frontal Orbital Cortex |  | 20.70 | 34 | 28 | 2 |
|  | R Frontal Operculum Cortex |  | 19.68 | 36 | 22 | 8 |
|  | R Central Opercular Cortex |  | 18.92 | 48 | 6 | 0 |
|  | R Juxtapositional Lobule Cortex |  | 18.84 | 6 | 6 | 48 |
|  | R Putamen |  | 18.23 | 22 | 6 | -6 |
|  | R Cingulate Gyrus, anterior division |  | 18.09 | 10 | 6 | 42 |
|  | R Precentral Gyrus |  | 18.04 | 44 | -2 | 48 |
|  | R Supramarginal Gyrus, posterior division |  | 17.55 | 62 | -42 | 22 |
|  | R Brain Stem |  | 17.44 | 4 | -30 | -2 |
|  | R Superior Frontal Gyrus |  | 17.16 | 2 | 10 | 56 |
|  | R Caudate |  | 17.06 | 12 | -2 | 14 |
|  | R Inferior Frontal Gyrus, pars triangularis |  | 16.12 | 56 | 22 | 0 |
|  | R Cingulate Gyrus, posterior division |  | 15.85 | 10 | -22 | 42 |
|  | R Parietal Operculum Cortex |  | 15.71 | 56 | -28 | 26 |
|  | R Frontal Pole |  | 15.62 | 32 | 48 | 28 |
|  | R Middle Frontal Gyrus |  | 14.95 | 50 | 8 | 42 |
|  | R Temporal Occipital Fusiform Cortex |  | 14.92 | 32 | -54 | -20 |
|  | R Superior Parietal Lobule |  | 14.40 | 24 | -46 | 66 |
|  | R Middle Temporal Gyrus, posterior division |  | 13.51 | 50 | -24 | -6 |
|  | R Angular Gyrus |  | 12.85 | 58 | -50 | 42 |
|  | R Thalamus |  | 12.81 | 6 | -20 | -2 |
|  | R Postcentral Gyrus |  | 12.03 | 32 | -38 | 62 |
|  | R Inferior Frontal Gyrus, pars opercularis |  | 11.81 | 40 | 16 | 26 |
|  | R Precuneus Cortex |  | 11.27 | 8 | -54 | 56 |
|  | R Middle Temporal Gyrus, temporooccipital part |  | 11.23 | 54 | -60 | 6 |
|  | R Occipital Pole |  | 11.13 | 22 | -96 | 16 |
|  | R Lingual Gyrus |  | 9.26 | 10 | -80 | -6 |
|  | R Planum Polare |  | 9.15 | 42 | -4 | -12 |
|  | R Amygdala |  | 9.14 | 28 | -10 | -12 |
|  | R Occipital Fusiform Gyrus |  | 8.91 | 22 | -80 | -12 |
|  | R Lateral Occipital Cortex, inferior division |  | 8.83 | 42 | -78 | -10 |
|  | R Lateral Occipital Cortex, superior division |  | 8.58 | 16 | -80 | 40 |
|  | R Insular Cortex |  | 8.33 | 40 | -16 | -2 |
|  | R Pallidum |  | 7.30 | 14 | -4 | -4 |
|  | R Inferior Temporal Gyrus, temporooccipital part |  | 7.25 | 48 | -54 | -14 |
|  | R Heschls Gyrus (includes H1 and H2) |  | 6.53 | 44 | -12 | 4 |
|  | **Cluster 2** | 15,144 |  |  |  |  |
|  | L Frontal Pole |  | 16.21 | -28 | 48 | 26 |
|  | L Middle Frontal Gyrus |  | 14.75 | -34 | 36 | 40 |
|  | **Cluster 3** | 1,672 |  |  |  |  |
|  | R Temporal Pole |  | 6.08 | 46 | 12 | -42 |
|  | **Cluster 4** | 688 |  |  |  |  |
|  | L Middle Temporal Gyrus, posterior division |  | 6.27 | -60 | -28 | -2 |
|  | **Cluster 5** | 376 |  |  |  |  |
|  | R Frontal Pole |  | 7.49 | 26 | 50 | -14 |
|  | **Cluster 6** | 320 |  |  |  |  |
|  | L Temporal Pole |  | 6.89 | -40 | 6 | -38 |
|  | **Cluster 7** | 272 |  |  |  |  |
|  | L Middle Temporal Gyrus, anterior division |  | 6.50 | -50 | -2 | -30 |
|  | **Cluster 8** | 216 |  |  |  |  |
|  | R Occipital Pole |  | 6.74 | 6 | -96 | -8 |
|  | **Cluster 9** | 176 |  |  |  |  |
|  | R Temporal Pole |  | 6.72 | 52 | 8 | -20 |
|  | **Cluster 10** | 144 |  |  |  |  |
|  | R Parahippocampal Gyrus, anterior division |  | 6.19 | 24 | -8 | -32 |
|  | **Cluster 11** | 104 |  |  |  |  |
|  | L Amygdala |  | 7.67 | -26 | -12 | -12 |
|  | **Cluster 12** | 80 |  |  |  |  |
|  | L Frontal Pole |  | 6.38 | -30 | 54 | -14 |
|  | **Cluster 13** | 64 |  |  |  |  |
|  | L Parahippocampal Gyrus, anterior division |  | 5.89 | -24 | -6 | -32 |
|  | **Cluster 14** | 24 |  |  |  |  |
|  | L Temporal Fusiform Cortex, anterior division |  | 5.22 | -36 | -2 | -42 |
|  | **Cluster 15** | 24 |  |  |  |  |
|  | L Frontal Pole |  | 5.67 | -26 | 46 | -14 |
|  | **Cluster 16** | 24 |  |  |  |  |
|  | R Frontal Pole |  | 5.55 | 30 | 66 | 0 |
|  | **Cluster 17** | 16 |  |  |  |  |
|  | L Parahippocampal Gyrus, anterior division |  | 5.58 | -24 | -10 | -38 |
|  | **Cluster 18** | 16 |  |  |  |  |
|  | R Amygdala |  | 5.53 | 18 | -14 | -12 |
|  | **Cluster 19** | 8 |  |  |  |  |
|  | L Temporal Pole |  | 5.24 | -20 | 2 | -40 |
|  | **Cluster 20** | 8 |  |  |  |  |
|  | R Brain Stem |  | 5.70 | 8 | -24 | -34 |
|  | **Cluster 21** | 8 |  |  |  |  |
|  | R Hippocampus |  | 5.44 | 12 | -12 | -20 |
|  | **Cluster 22** | 8 |  |  |  |  |
|  | L Frontal Pole |  | 5.14 | -24 | 50 | -14 |
|  | **Cluster 23** | 8 |  |  |  |  |
|  | R Frontal Pole |  | 5.13 | 22 | 62 | -8 |
|  | **Cluster 24** | 8 |  |  |  |  |
|  | R Hippocampus |  | 5.15 | 34 | -28 | -6 |
|  | **Cluster 25** | 8 |  |  |  |  |
|  | L Frontal Pole |  | 5.34 | -26 | 66 | -4 |
|  | **Cluster 26** | 8 |  |  |  |  |
|  | L Hippocampus |  | 5.20 | -20 | -38 | -2 |
|  | **Cluster 27** | 8 |  |  |  |  |
|  | R Frontal Pole |  | 5.36 | 14 | 64 | 26 |
|  | **Cluster 28** | 8 |  |  |  |  |
|  | R Postcentral Gyrus |  | 5.42 | 22 | -30 | 62 |

Note: Suprathreshold activation was also evident in the bilateral periaqueductal gray, bilateral bed nucleus of the stria terminalis, and bilateral dorsal amygdala. 

**Supplementary Table S2.** Descriptive statistics for clusters and local extrema showing greater activation during the anticipation of Uncertain Safety relative to Uncertain Threat (FWE *p*<0.05, whole-brain corrected, 4-mm smoothing kernel).

|  |  | **mm**^**3**^ | ***t*** | **x** | **y** | **z** |
| --- | --- | --- | --- | --- | --- | --- |
|  | **Cluster 1** | 30,088 |  |  |  |  |
|  | L Intracalcarine Cortex |  | 19.11 | -14 | -80 | 6 |
|  | L Cingulate Gyrus, posterior division |  | 10.74 | -4 | -50 | 14 |
|  | L Lingual Gyrus |  | 10.55 | -10 | -60 | 4 |
|  | L Precuneus Cortex |  | 10.40 | -10 | -62 | 18 |
|  | R Intracalcarine Cortex |  | 18.87 | 14 | -78 | 10 |
|  | R Precuneus Cortex |  | 14.57 | 2 | -68 | 22 |
|  | R Supracalcarine Cortex |  | 13.91 | 2 | -66 | 18 |
|  | R Cingulate Gyrus, posterior division |  | 10.39 | 10 | -50 | 4 |
|  | **Cluster 2** | 5,000 |  |  |  |  |
|  | L Postcentral Gyrus |  | 11.41 | -52 | -10 | 26 |
|  | **Cluster 3** | 3,640 |  |  |  |  |
|  | L Precentral Gyrus |  | 8.43 | -6 | -24 | 60 |
|  | L Postcentral Gyrus |  | 6.85 | -10 | -40 | 70 |
|  | R Precentral Gyrus |  | 10.41 | 2 | -32 | 66 |
|  | **Cluster 4** | 3,112 |  |  |  |  |
|  | R Precentral Gyrus |  | 11.07 | 54 | -6 | 26 |
|  | R Postcentral Gyrus |  | 9.93 | 64 | -6 | 34 |
|  | **Cluster 5** | 2,600 |  |  |  |  |
|  | L Frontal Medial Cortex |  | 5.73 | -6 | 54 | -12 |
|  | R Frontal Pole |  | 9.58 | 0 | 64 | -10 |
|  | R Frontal Medial Cortex |  | 8.36 | 2 | 52 | -12 |
|  | R Subcallosal Cortex |  | 5.49 | 4 | 28 | -18 |
|  | **Cluster 6** | 1,568 |  |  |  |  |
|  | L Lateral Occipital Cortex, superior division |  | 7.85 | -38 | -84 | 38 |
|  | **Cluster 7** | 1,328 |  |  |  |  |
|  | R Lateral Occipital Cortex, superior division |  | 7.61 | 50 | -72 | 36 |
|  | **Cluster 8** | 1,016 |  |  |  |  |
|  | L Insular Cortex |  | 10.02 | -38 | -12 | 14 |
|  | L Central Opercular Cortex |  | 7.88 | -44 | -12 | 18 |
|  | **Cluster 9** | 872 |  |  |  |  |
|  | R Insular Cortex |  | 10.33 | 38 | -8 | 12 |
|  | R Central Opercular Cortex |  | 7.57 | 46 | -10 | 16 |
|  | **Cluster 10** | 576 |  |  |  |  |
|  | R Parahippocampal Gyrus, posterior division |  | 7.93 | 28 | -32 | -18 |
|  | R Lingual Gyrus |  | 6.21 | 26 | -40 | -10 |
|  | **Cluster 11** | 568 |  |  |  |  |
|  | L Parahippocampal Gyrus, posterior division |  | 7.43 | -28 | -38 | -14 |
|  | L Temporal Fusiform Cortex, posterior division |  | 6.31 | -32 | -32 | -18 |
|  | **Cluster 12** | 328 |  |  |  |  |
|  | R Precentral Gyrus |  | 6.19 | 14 | -28 | 66 |
|  | **Cluster 13** | 248 |  |  |  |  |
|  | R Hippocampus |  | 7.49 | 22 | -18 | -20 |
|  | R Amygdala |  | 5.51 | 18 | -8 | -18 |
|  | **Cluster 14** | 184 |  |  |  |  |
|  | L Hippocampus |  | 7.83 | -18 | -16 | -22 |
|  | **Cluster 15** | 112 |  |  |  |  |
|  | R Postcentral Gyrus |  | 6.93 | 12 | -40 | 70 |
|  | **Cluster 16** | 88 |  |  |  |  |
|  | L Cingulate Gyrus, posterior division |  | 5.62 | -6 | -38 | 34 |
|  | **Cluster 17** | 88 |  |  |  |  |
|  | L Precentral Gyrus |  | 6.61 | -12 | -28 | 72 |
|  | **Cluster 18** | 80 |  |  |  |  |
|  | R Frontal Pole |  | 5.91 | 38 | 36 | -12 |
|  | **Cluster 19** | 56 |  |  |  |  |
|  | L Middle Temporal Gyrus, anterior division |  | 5.94 | -64 | -4 | -18 |
|  | **Cluster 20** | 56 |  |  |  |  |
|  | R Superior Frontal Gyrus |  | 5.32 | 20 | 30 | 46 |
|  | **Cluster 21** | 24 |  |  |  |  |
|  | L Hippocampus |  | 5.48 | -22 | -20 | -16 |
|  | **Cluster 22** | 24 |  |  |  |  |
|  | L Frontal Orbital Cortex |  | 5.59 | -34 | 36 | -10 |
|  | **Cluster 23** | 24 |  |  |  |  |
|  | R Lingual Gyrus |  | 5.38 | 18 | -46 | -8 |
|  | **Cluster 24** | 24 |  |  |  |  |
|  | R Subcallosal Cortex |  | 5.79 | 2 | 14 | -6 |
|  | **Cluster 25** | 16 |  |  |  |  |
|  | R Middle Temporal Gyrus, anterior division |  | 5.55 | 64 | 0 | -20 |
|  | **Cluster 26** | 16 |  |  |  |  |
|  | L Precentral Gyrus |  | 5.18 | -12 | -26 | 78 |
|  | **Cluster 27** | 8 |  |  |  |  |
|  | R Subcallosal Cortex |  | 5.21 | 2 | 22 | -24 |
|  | **Cluster 28** | 8 |  |  |  |  |
|  | R Subcallosal Cortex |  | 5.19 | 0 | 26 | -24 |
|  | **Cluster 29** | 8 |  |  |  |  |
|  | L Amygdala |  | 5.32 | -30 | 2 | -18 |
|  | **Cluster 30** | 8 |  |  |  |  |
|  | L Hippocampus |  | 5.38 | -28 | -18 | -16 |
|  | **Cluster 31** | 8 |  |  |  |  |
|  | L Frontal Orbital Cortex |  | 5.11 | -40 | 36 | -14 |
|  | **Cluster 32** | 8 |  |  |  |  |
|  | L Frontal Medial Cortex |  | 5.11 | -6 | 48 | -14 |
|  | **Cluster 33** | 8 |  |  |  |  |
|  | L Frontal Medial Cortex |  | 5.19 | -8 | 52 | -10 |
|  | **Cluster 34** | 8 |  |  |  |  |
|  | R Hippocampus |  | 5.72 | 24 | -26 | -8 |
|  | **Cluster 35** | 8 |  |  |  |  |
|  | R Cuneal Cortex |  | 5.12 | 0 | -86 | 34 |

**Supplementary Table S3.** Descriptive statistics for clusters and local extrema showing greater activation during the anticipation of Certain Threat relative to Certain Safety (FWE *p*<0.05, whole-brain corrected). 

|  |  | **mm**^**3**^ | ***t*** | **x** | **y** | **z** |
| --- | --- | --- | --- | --- | --- | --- |
|  | **Cluster 1** | 448,752 |  |  |  |  |
|  | L Superior Frontal Gyrus |  | 15.05 | -14 | -2 | 68 |
|  | L Frontal Operculum Cortex |  | 14.25 | -34 | 16 | 10 |
|  | L Putamen |  | 13.61 | -22 | 14 | -6 |
|  | L Cingulate Gyrus, anterior division |  | 13.34 | -8 | 18 | 34 |
|  | L Juxtapositional Lobule Cortex |  | 13.24 | -4 | 4 | 56 |
|  | L Thalamus |  | 13.19 | -10 | -6 | 14 |
|  | L Bed Nucleus of the Stria Terminalis |  | 12.97 | -6 | 4 | 0 |
|  | L Supramarginal Gyrus, posterior division |  | 12.80 | -58 | -50 | 40 |
|  | L Paracingulate Gyrus |  | 12.72 | -8 | 22 | 32 |
|  | L Lateral Occipital Cortex, superior division |  | 12.56 | -36 | -58 | 58 |
|  | L Middle Frontal Gyrus |  | 12.05 | -34 | -4 | 64 |
|  | L Insular Cortex |  | 11.78 | -32 | 26 | 4 |
|  | L Superior Parietal Lobule |  | 11.68 | -34 | -58 | 52 |
|  | L Occipital Fusiform Gyrus |  | 11.61 | -30 | -72 | -20 |
|  | L Brain Stem |  | 11.52 | -2 | -28 | -4 |
|  | L Frontal Pole |  | 11.51 | -30 | 52 | 28 |
|  | L Lingual Gyrus |  | 11.30 | -4 | -74 | -12 |
|  | L Central Opercular Cortex |  | 11.20 | -44 | 4 | 2 |
|  | L Caudate |  | 10.65 | -14 | 18 | -4 |
|  | L Precentral Gyrus |  | 10.62 | -40 | -2 | 46 |
|  | L Frontal Orbital Cortex |  | 10.50 | -32 | 26 | -6 |
|  | L Inferior Frontal Gyrus, pars triangularis |  | 10.16 | -44 | 22 | 8 |
|  | L Angular Gyrus |  | 9.90 | -50 | -56 | 48 |
|  | L Parietal Operculum Cortex |  | 9.77 | -60 | -38 | 24 |
|  | L Precuneus Cortex |  | 9.49 | -10 | -78 | 36 |
|  | L Cingulate Gyrus, posterior division |  | 9.31 | -2 | -26 | 28 |
|  | L Supramarginal Gyrus, anterior division |  | 8.34 | -60 | -38 | 46 |
|  | L Postcentral Gyrus |  | 8.30 | -20 | -32 | 76 |
|  | L Accumbens |  | 7.92 | -8 | 12 | -6 |
|  | L Inferior Frontal Gyrus, pars opercularis |  | 7.69 | -54 | 10 | 16 |
|  | L Middle Temporal Gyrus, temporooccipital part |  | 7.01 | -62 | -56 | 6 |
|  | L Lateral Occipital Cortex, inferior division |  | 6.25 | -56 | -64 | 10 |
|  | L Temporal Pole |  | 6.12 | -50 | 18 | -18 |
|  | L Hippocampus |  | 5.64 | -30 | -12 | -16 |
|  | L Periaqueductal Gray |  | 5.52 | -2 | -34 | -12 |
|  | L Superior Temporal Gyrus, posterior division |  | 5.30 | -66 | -38 | 6 |
|  | R Putamen |  | 15.93 | 22 | 10 | -8 |
|  | R Superior Frontal Gyrus |  | 15.64 | 20 | -8 | 70 |
|  | R Paracingulate Gyrus |  | 15.16 | 8 | 20 | 36 |
|  | R Caudate |  | 14.92 | 12 | -4 | 16 |
|  | R Precentral Gyrus |  | 14.52 | 46 | 0 | 50 |
|  | R Juxtapositional Lobule Cortex |  | 14.08 | 2 | 6 | 48 |
|  | R Bed Nucleus of the Stria Terminalis |  | 13.93 | 8 | 4 | 2 |
|  | R Postcentral Gyrus |  | 13.84 | 34 | -36 | 62 |
|  | R Thalamus |  | 13.41 | 10 | -2 | 12 |
|  | R Frontal Pole |  | 13.02 | 30 | 44 | 24 |
|  | R Superior Parietal Lobule |  | 12.93 | 24 | -44 | 66 |
|  | R Frontal Operculum Cortex |  | 12.55 | 34 | 24 | 8 |
|  | R Supramarginal Gyrus, posterior division |  | 12.54 | 62 | -38 | 32 |
|  | R Middle Frontal Gyrus |  | 12.45 | 38 | -2 | 50 |
|  | R Angular Gyrus |  | 12.29 | 62 | -46 | 30 |
|  | R Central Opercular Cortex |  | 11.69 | 52 | 6 | 2 |
|  | R Lateral Occipital Cortex, superior division |  | 11.46 | 28 | -58 | 62 |
|  | R Brain Stem |  | 10.80 | 4 | -28 | -2 |
|  | R Superior Temporal Gyrus, posterior division |  | 10.67 | 48 | -26 | -4 |
|  | R Cingulate Gyrus, anterior division |  | 10.16 | 0 | 10 | 34 |
|  | R Precuneus Cortex |  | 10.01 | 8 | -56 | 56 |
|  | R Frontal Orbital Cortex |  | 9.63 | 40 | 20 | -8 |
|  | R Cingulate Gyrus, posterior division |  | 9.54 | 10 | -24 | 42 |
|  | R Accumbens |  | 9.49 | 10 | 12 | -4 |
|  | R Parietal Operculum Cortex |  | 9.06 | 56 | -28 | 26 |
|  | R Inferior Frontal Gyrus, pars triangularis |  | 8.66 | 54 | 28 | -6 |
|  | R Supramarginal Gyrus, anterior division |  | 8.36 | 60 | -24 | 28 |
|  | R Middle Temporal Gyrus, posterior division |  | 8.32 | 54 | -22 | -10 |
|  | R Amygdala |  | 8.32 | 30 | -10 | -14 |
|  | R Inferior Frontal Gyrus, pars opercularis |  | 7.95 | 46 | 12 | 28 |
|  | R Lateral Occipital Cortex, inferior division |  | 7.74 | 42 | -84 | -8 |
|  | R Middle Temporal Gyrus, temporooccipital part |  | 7.32 | 60 | -60 | 6 |
|  | R Insular Cortex |  | 6.99 | 40 | 10 | -8 |
|  | R Planum Polare |  | 6.26 | 42 | -4 | -14 |
|  | R Occipital Pole |  | 5.88 | 26 | -94 | 18 |
|  | R Pallidum |  | 5.47 | 16 | -4 | -4 |
|  | R Periaqueductal Gray |  | 5.40 | 2 | -36 | -12 |
|  | R Temporal Occipital Fusiform Cortex |  | 5.30 | 44 | -54 | -16 |
|  | **Cluster 2** | 792 |  |  |  |  |
|  | L Brain Stem |  | 6.73 | -8 | -38 | -46 |
|  | R Brain Stem |  | 8.04 | 2 | -34 | -48 |
|  | **Cluster 3** | 776 |  |  |  |  |
|  | L Lateral Occipital Cortex, inferior division |  | 7.00 | -40 | -82 | -6 |
|  | L Occipital Pole |  | 5.50 | -36 | -92 | -8 |
|  | **Cluster 4** |  |  |  |  |  |
|  | Middle Temporal Gyrus, posterior division |  | 6.95 | -66 | -24 | -6 |
|  | Superior Temporal Gyrus, posterior division |  | 6.71 | -60 | -26 | -2 |
|  | **Cluster 5** | 224 |  |  |  |  |
|  | R Temporal Pole |  | 6.31 | 42 | 6 | -38 |
|  | R Middle Temporal Gyrus, anterior division |  | 6.29 | 48 | 0 | -32 |
|  | **Cluster 6** | 224 |  |  |  |  |
|  | L Frontal Pole |  | 6.05 | -32 | 48 | -14 |
|  | **Cluster 7** | 168 |  |  |  |  |
|  | L Occipital Pole |  | 5.82 | -22 | -96 | 12 |
|  | **Cluster 8** | 168 |  |  |  |  |
|  | R Precentral Gyrus |  | 6.85 | 6 | -30 | 76 |
|  | **Cluster 9** | 152 |  |  |  |  |
|  | R Frontal Pole |  | 6.34 | 40 | 46 | 6 |
|  | **Cluster 10** | 96 |  |  |  |  |
|  | L Hippocampus |  | 6.17 | -22 | -36 | -4 |
|  | **Cluster 11** |  |  |  |  |  |
|  | L Planum Polare |  | 6.74 | -42 | -4 | -14 |
|  | L Insular Cortex |  | 6.58 | -40 | -2 | -16 |
|  | **Cluster 12** | 88 |  |  |  |  |
|  | R Frontal Pole |  | 6.05 | 26 | 48 | -14 |
|  | **Cluster 13** | 80 |  |  |  |  |
|  | R Brain Stem |  | 5.97 | 18 | -34 | -28 |
|  | **Cluster 14** | 72 |  |  |  |  |
|  | L Lateral Occipital Cortex, inferior division |  | 5.73 | -48 | -64 | 4 |
|  | **Cluster 15** | 72 |  |  |  |  |
|  | L Middle Temporal Gyrus, temporooccipital part |  | 6.13 | -46 | -58 | 10 |
|  | **Cluster 16** | 64 |  |  |  |  |
|  | L Brain Stem |  | 6.46 | -6 | -46 | -56 |
|  | **Cluster 17** | 64 |  |  |  |  |
|  | R Brain Stem |  | 5.58 | 0 | -36 | -38 |
|  | **Cluster 18** | 64 |  |  |  |  |
|  | L Brain Stem |  | 6.95 | -4 | -34 | -24 |
|  | **Cluster 19** | 48 |  |  |  |  |
|  | L Brain Stem |  | 6.79 | -12 | -24 | -20 |
|  | **Cluster 20** | 48 |  |  |  |  |
|  | L Lateral Occipital Cortex, inferior division |  | 5.54 | -50 | -74 | -10 |
|  | **Cluster 21** | 40 |  |  |  |  |
|  | L Brain Stem |  | 5.71 | -8 | -38 | -30 |
|  | **Cluster 22** | 40 |  |  |  |  |
|  | L Brain Stem |  | 5.71 | -8 | -20 | -22 |
|  | **Cluster 23** | 32 |  |  |  |  |
|  | L Brain Stem |  | 6.15 | -4 | -18 | -20 |
|  | **Cluster 24** | 32 |  |  |  |  |
|  | R Inferior Temporal Gyrus, temporooccipital part |  | 5.86 | 60 | -58 | -18 |
|  | **Cluster 25** | 32 |  |  |  |  |
|  | R Inferior Temporal Gyrus, temporooccipital part |  | 5.50 | 60 | -58 | -12 |
|  | **Cluster 26** | 32 |  |  |  |  |
|  | R Lateral Occipital Cortex, inferior division |  | 5.44 | 44 | -72 | -4 |
|  | **Cluster 27** | 32 |  |  |  |  |
|  | R Hippocampus |  | 5.63 | 20 | -36 | 6 |
|  | **Cluster 28** | 32 |  |  |  |  |
|  | R Central Opercular Cortex |  | 5.32 | 48 | -18 | 14 |
|  | **Cluster 29** | 16 |  |  |  |  |
|  | R Hippocampus |  | 5.95 | 16 | -12 | -16 |
|  | **Cluster 30** | 16 |  |  |  |  |
|  | R Cingulate Gyrus, posterior division |  | 5.44 | 4 | -38 | 24 |
|  | **Cluster 31** | 16 |  |  |  |  |
|  | L Middle Frontal Gyrus |  | 5.24 | -50 | 22 | 30 |
|  | **Cluster 32** | 8 |  |  |  |  |
|  | R Brain Stem |  | 5.20 | 8 | -36 | -28 |
|  | **Cluster 33** | 8 |  |  |  |  |
|  | R Brain Stem |  | 5.25 | 6 | -34 | -26 |
|  | **Cluster 34** | 8 |  |  |  |  |
|  | L Middle Temporal Gyrus, posterior division |  | 5.17 | -54 | -30 | -6 |
|  | **Cluster 35** | 8 |  |  |  |  |
|  | L Pallidum |  | 5.25 | -20 | -10 | -4 |
|  | **Cluster 36** | 8 |  |  |  |  |
|  | L Frontal Pole |  | 5.26 | -50 | 42 | 0 |
|  | **Cluster 37** | 8 |  |  |  |  |
|  | R Frontal Pole |  | 5.98 | 30 | 68 | 2 |
|  | **Cluster 38** | 8 |  |  |  |  |
|  | L Lateral Occipital Cortex, inferior division |  | 5.43 | -54 | -70 | 6 |
|  | **Cluster 39** | 8 |  |  |  |  |
|  | L Lateral Occipital Cortex, inferior division |  | 5.52 | -42 | -72 | 10 |
|  | **Cluster 40** | 8 |  |  |  |  |
|  | R Lateral Occipital Cortex, inferior division |  | 6.02 | 42 | -68 | 10 |
|  | **Cluster 41** | 8 |  |  |  |  |
|  | R Lateral Occipital Cortex, inferior division |  | 5.24 | 42 | -62 | 12 |
|  | **Cluster 42** | 8 |  |  |  |  |
|  | R Parietal Operculum Cortex |  | 5.20 | 46 | -22 | 16 |
|  | **Cluster 43** | 8 |  |  |  |  |
|  | R Occipital Pole |  | 5.22 | 18 | -98 | 18 |
|  | **Cluster 44** | 8 |  |  |  |  |
|  | L Angular Gyrus |  | 5.35 | -58 | -60 | 18 |
|  | **Cluster 45** | 8 |  |  |  |  |
|  | R Inferior Frontal Gyrus, pars triangularis |  | 5.40 | 50 | 26 | 18 |
|  | **Cluster 46** | 8 |  |  |  |  |
|  | R Lateral Occipital Cortex, superior division |  | 5.26 | 26 | -84 | 36 |

Note: Suprathreshold activation was also evident in the bilateral dorsal amygdala.

**Supplementary Table S4.** Descriptive statistics for clusters and local extrema showing greater activation during the anticipation of Certain Safety relative to Certain Threat (FWE *p*<0.05, whole-brain corrected, 4-mm smoothing kernel).

|  |  | **mm**^**3**^ | ***t*** | **x** | **y** | **z** |
| --- | --- | --- | --- | --- | --- | --- |
|  | **Cluster 1** | 35,472 |  |  |  |  |
|  | L Intracalcarine Cortex |  | 19.18 | -10 | -76 | 10 |
|  | L Lingual Gyrus |  | 10.42 | -20 | -44 | -10 |
|  | L Precuneus Cortex |  | 8.68 | -6 | -60 | 12 |
|  | L Occipital Pole |  | 8.46 | -10 | -98 | -4 |
|  | L Parahippocampal Gyrus, posterior division |  | 7.48 | -30 | -34 | -16 |
|  | L Cingulate Gyrus, posterior division |  | 6.42 | -4 | -48 | 14 |
|  | R Intracalcarine Cortex |  | 20.15 | 14 | -82 | 4 |
|  | R Lingual Gyrus |  | 17.44 | 0 | -74 | 6 |
|  | R Occipital Pole |  | 15.03 | 6 | -90 | 6 |
|  | R Precuneus Cortex |  | 13.09 | 0 | -68 | 18 |
|  | R Parahippocampal Gyrus, posterior division |  | 8.09 | 20 | -34 | -16 |
|  | R Occipital Fusiform Gyrus |  | 8.01 | 28 | -68 | -6 |
|  | R Cingulate Gyrus, posterior division |  | 7.98 | 10 | -50 | 6 |
|  | R Temporal Fusiform Cortex, posterior division |  | 7.67 | 30 | -34 | -18 |
|  | **Cluster 2** | 288 |  |  |  |  |
|  | L Postcentral Gyrus |  | 6.49 | -62 | -8 | 22 |
|  | **Cluster 3** | 272 |  |  |  |  |
|  | L Subcallosal Cortex |  | 6.25 | -2 | 16 | -6 |
|  | R Subcallosal Cortex |  | 7.09 | 2 | 14 | -10 |
|  | **Cluster 4** | 168 |  |  |  |  |
|  | R Frontal Medial Cortex |  | 5.70 | 2 | 44 | -14 |
|  | **Cluster 5** | 160 |  |  |  |  |
|  | L Lingual Gyrus |  | 6.47 | -28 | -58 | -6 |
|  | **Cluster 6** | 40 |  |  |  |  |
|  | R Frontal Medial Cortex |  | 5.48 | 0 | 50 | -14 |
|  | **Cluster 7** | 32 |  |  |  |  |
|  | L Temporal Pole |  | 5.58 | -42 | 12 | -18 |
|  | **Cluster 8** | 32 |  |  |  |  |
|  | R Subcallosal Cortex |  | 5.56 | 0 | 30 | -16 |
|  | **Cluster 9** | 32 |  |  |  |  |
|  | R Precentral Gyrus |  | 5.34 | 56 | -6 | 24 |
|  | **Cluster 10** | 24 |  |  |  |  |
|  | R Parahippocampal Gyrus, anterior division |  | 5.94 | 20 | -20 | -22 |
|  | **Cluster 11** | 16 |  |  |  |  |
|  | L Frontal Medial Cortex |  | 5.54 | -2 | 36 | -18 |
|  | **Cluster 12** | 16 |  |  |  |  |
|  | R Right Hippocampus |  | 6.65 | 24 | -26 | -8 |
|  | **Cluster 13** | 16 |  |  |  |  |
|  | R Precentral Gyrus |  | 5.39 | 60 | -4 | 28 |
|  | **Cluster 14** | 8 |  |  |  |  |
|  | L Middle Temporal Gyrus, anterior division |  | 5.29 | -56 | -6 | -16 |
|  | **Cluster 15** | 8 |  |  |  |  |
|  | L Frontal Medial Cortex |  | 5.16 | -8 | 42 | -14 |
|  | **Cluster 16** | 8 |  |  |  |  |
|  | L Frontal Pole |  | 5.15 | -4 | 56 | -12 |
|  | **Cluster 17** | 8 |  |  |  |  |
|  | R Postcentral Gyrus |  | 5.44 | 66 | -6 | 22 |

**Supplementary Table S5.** Descriptive statistics for clusters and local extrema showing greater activation during the anticipation of Uncertain Threat relative to Certain Threat (FWE *p*<0.05, whole-brain corrected).

|  |  | **mm**^**3**^ | ***t*** | **x** | **y** | **z** |
| --- | --- | --- | --- | --- | --- | --- |
|  | **Cluster 1** | 142,448 |  |  |  |  |
|  | L Occipital Pole |  | 13.58 | -12 | -94 | -8 |
|  | L Temporal Occipital Fusiform Cortex |  | 11.63 | -24 | -54 | -16 |
|  | L Occipital Fusiform Gyrus |  | 11.36 | -20 | -86 | -10 |
|  | L Cingulate Gyrus, anterior division |  | 9.84 | -6 | 8 | 40 |
|  | L Lateral Occipital Cortex, inferior division |  | 9.30 | -32 | -86 | -18 |
|  | L Superior Frontal Gyrus |  | 8.75 | -14 | 10 | 66 |
|  | L Paracingulate Gyrus |  | 8.64 | -6 | 28 | 30 |
|  | L Temporal Fusiform Cortex, posterior division |  | 5.82 | -42 | -40 | -24 |
|  | R Occipital Fusiform Gyrus |  | 15.81 | 30 | -78 | -10 |
|  | R Frontal Orbital Cortex |  | 15.80 | 34 | 28 | 2 |
|  | R Frontal Operculum Cortex |  | 15.11 | 42 | 24 | 4 |
|  | R Occipital Pole |  | 14.00 | 26 | -94 | 12 |
|  | R Precentral Gyrus |  | 13.40 | 42 | -2 | 52 |
|  | R Lateral Occipital Cortex, inferior division |  | 13.31 | 44 | -80 | -10 |
|  | R Central Opercular Cortex |  | 12.41 | 56 | 4 | 4 |
|  | R Temporal Occipital Fusiform Cortex |  | 12.37 | 36 | -46 | -18 |
|  | R Middle Frontal Gyrus |  | 12.06 | 42 | 0 | 60 |
|  | R Inferior Frontal Gyrus, pars triangularis |  | 12.02 | 54 | 22 | 2 |
|  | R Parietal Operculum Cortex |  | 12.00 | 54 | -28 | 26 |
|  | R Juxtapositional Lobule Cortex |  | 11.96 | 4 | 4 | 46 |
|  | R Supramarginal Gyrus, anterior division |  | 11.81 | 60 | -26 | 24 |
|  | R Cingulate Gyrus, anterior division |  | 11.78 | 8 | 6 | 42 |
|  | R Paracingulate Gyrus |  | 11.62 | 2 | 18 | 38 |
|  | R Superior Frontal Gyrus |  | 10.58 | 4 | 20 | 58 |
|  | R Inferior Frontal Gyrus, pars opercularis |  | 10.43 | 60 | 12 | 8 |
|  | R Supramarginal Gyrus, posterior division |  | 10.37 | 58 | -44 | 30 |
|  | R Putamen |  | 10.28 | 28 | 2 | -6 |
|  | R Cingulate Gyrus, posterior division |  | 10.02 | 12 | -22 | 42 |
|  | R Angular Gyrus |  | 9.92 | 62 | -48 | 34 |
|  | R Middle Temporal Gyrus, temporooccipital part |  | 8.46 | 46 | -56 | 6 |
|  | R Inferior Temporal Gyrus, temporooccipital part |  | 6.80 | 46 | -42 | -16 |
|  | R Planum Polare |  | 6.49 | 40 | -18 | -2 |
|  | R Frontal Pole |  | 6.14 | 54 | 38 | -8 |
|  | R Insular Cortex |  | 5.73 | 38 | -14 | 4 |
|  | R Pallidum |  | 5.49 | 16 | 2 | 0 |
|  | R Middle Temporal Gyrus, posterior division |  | 5.36 | 56 | -34 | 0 |
|  | **Cluster 2** | 13,728 |  |  |  |  |
|  | L Insular Cortex |  | 16.18 | -32 | 24 | 6 |
|  | L Frontal Operculum Cortex |  | 14.44 | -44 | 20 | -4 |
|  | L Inferior Frontal Gyrus, pars opercularis |  | 10.78 | -50 | 20 | 4 |
|  | L Central Opercular Cortex |  | 10.68 | -56 | 2 | 4 |
|  | L Precentral Gyrus |  | 8.83 | -56 | 8 | 2 |
|  | **Cluster 3** | 4,056 |  |  |  |  |
|  | L Supramarginal Gyrus, posterior division |  | 8.05 | -60 | -50 | 44 |
|  | L Supramarginal Gyrus, anterior division |  | 8.04 | -64 | -32 | 22 |
|  | L Parietal Operculum Cortex |  | 7.77 | -62 | -30 | 20 |
|  | L Angular Gyrus |  | 7.19 | -60 | -54 | 38 |
|  | **Cluster 4** | 2,272 |  |  |  |  |
|  | R Superior Parietal Lobule |  | 10.02 | 22 | -46 | 66 |
|  | R Postcentral Gyrus |  | 6.80 | 34 | -38 | 62 |
|  | **Cluster 5** | 1,280 |  |  |  |  |
|  | L Frontal Pole |  | 7.03 | -38 | 46 | 30 |
|  | **Cluster 6** | 1,232 |  |  |  |  |
|  | L Brain Stem |  | 6.84 | -2 | -30 | -2 |
|  | R Brain Stem |  | 9.53 | 4 | -30 | -2 |
|  | R Thalamus |  | 7.70 | 6 | -22 | -2 |
|  | **Cluster 7** | 1,032 |  |  |  |  |
|  | R Thalamus |  | 7.98 | 10 | 0 | 8 |
|  | R Caudate |  | 6.20 | 16 | -10 | 20 |
|  | **Cluster 8** | 776 |  |  |  |  |
|  | R Frontal Pole |  | 6.68 | 32 | 50 | 26 |
|  | **Cluster 9** | 744 |  |  |  |  |
|  | L Putamen |  | 8.43 | -26 | 6 | -8 |
|  | L Insular Cortex |  | 6.78 | -36 | -2 | -6 |
|  | **Cluster 10** | 680 |  |  |  |  |
|  | L Precentral Gyrus |  | 7.07 | -36 | -4 | 48 |
|  | L Middle Frontal Gyrus |  | 6.44 | -38 | -2 | 56 |
|  | **Cluster 11** | 608 |  |  |  |  |
|  | R Middle Temporal Gyrus, posterior division |  | 7.23 | 52 | -22 | -6 |
|  | **Cluster 12** | 552 |  |  |  |  |
|  | L Cingulate Gyrus, posterior division |  | 6.42 | -2 | -16 | 28 |
|  | R Cingulate Gyrus, posterior division |  | 7.41 | 4 | -26 | 26 |
|  | **Cluster 13** | 216 |  |  |  |  |
|  | L Brain Stem |  | 7.06 | -6 | -36 | -48 |
|  | **Cluster 14** | 200 |  |  |  |  |
|  | L Superior Parietal Lobule |  | 6.31 | -18 | -52 | 64 |
|  | **Cluster 15** | 176 |  |  |  |  |
|  | L Cingulate Gyrus, posterior division |  | 7.15 | -12 | -24 | 40 |
|  | **Cluster 16** | 104 |  |  |  |  |
|  | L Brain Stem |  | 6.27 | -8 | -28 | -18 |
|  | **Cluster 17** | 104 |  |  |  |  |
|  | L Inferior Temporal Gyrus, temporooccipital part |  | 5.82 | -44 | -60 | -12 |
|  | **Cluster 18** | 72 |  |  |  |  |
|  | L Thalamus |  | 5.82 | -6 | -14 | -2 |
|  | **Cluster 19** | 40 |  |  |  |  |
|  | L Brain Stem |  | 6.29 | -6 | -32 | -8 |
|  | **Cluster 20** | 40 |  |  |  |  |
|  | L Lateral Occipital Cortex, superior division |  | 5.70 | -30 | -82 | 18 |
|  | **Cluster 21** | 24 |  |  |  |  |
|  | L Brain Stem |  | 5.77 | -4 | -36 | -30 |
|  | **Cluster 22** | 24 |  |  |  |  |
|  | L Middle Frontal Gyrus |  | 5.30 | -44 | 34 | 36 |
|  | **Cluster 23** | 16 |  |  |  |  |
|  | R Heschls Gyrus (includes H1 and H2) |  | 5.34 | 46 | -14 | 6 |
|  | **Cluster 24** | 16 |  |  |  |  |
|  | L Inferior Frontal Gyrus, pars opercularis |  | 5.19 | -56 | 12 | 18 |
|  | **Cluster 25** | 16 |  |  |  |  |
|  | R Cingulate Gyrus, anterior division |  | 5.37 | 8 | 38 | 18 |
|  | **Cluster 26** | 16 |  |  |  |  |
|  | R Lateral Occipital Cortex, superior division |  | 5.22 | 34 | -72 | 24 |
|  | **Cluster 27** | 8 |  |  |  |  |
|  | L Temporal Pole |  | 5.23 | -42 | 6 | -42 |
|  | **Cluster 28** | 8 |  |  |  |  |
|  | L Planum Polare |  | 5.51 | -40 | -20 | -4 |
|  | **Cluster 29** | 8 |  |  |  |  |
|  | R Insular Cortex |  | 5.20 | 36 | -22 | 6 |
|  | **Cluster 30** | 8 |  |  |  |  |
|  | R Angular Gyrus |  | 5.17 | 48 | -54 | 56 |

**Supplementary Table S6.** Descriptive statistics for clusters and local extrema showing greater activation during the anticipation of Certain Threat relative to Uncertain Threat (FWE *p*<0.05, whole-brain corrected, 4-mm smoothing kernel).

|  |  | **mm**^**3**^ | ***t*** | **x** | **y** | **z** |
| --- | --- | --- | --- | --- | --- | --- |
|  | **Cluster 1** | 35,560 |  |  |  |  |
|  | L Precuneus Cortex |  | 11.99 | -12 | -62 | 18 |
|  | L Lingual Gyrus |  | 11.19 | -12 | -54 | 0 |
|  | L Cingulate Gyrus, posterior division |  | 8.89 | -8 | -44 | 4 |
|  | L Hippocampus |  | 7.12 | -20 | -38 | 2 |
|  | L Thalamus |  | 6.87 | -14 | -36 | 4 |
|  | R Precuneus Cortex |  | 14.01 | 14 | -58 | 16 |
|  | R Cingulate Gyrus, posterior division |  | 11.01 | 10 | -48 | 4 |
|  | R Cuneal Cortex |  | 10.29 | 4 | -72 | 28 |
|  | R Lateral Occipital Cortex, superior division |  | 10.12 | 48 | -72 | 34 |
|  | R Hippocampus |  | 8.10 | 18 | -36 | 4 |
|  | **Cluster 2** | 21,344 |  |  |  |  |
|  | L Precentral Gyrus |  | 10.80 | -56 | -8 | 46 |
|  | L Postcentral Gyrus |  | 9.92 | -10 | -42 | 64 |
|  | R Postcentral Gyrus |  | 10.73 | 2 | -34 | 66 |
|  | R Precentral Gyrus |  | 8.82 | 6 | -22 | 66 |
|  | **Cluster 3** | 5,208 |  |  |  |  |
|  | L Lateral Occipital Cortex, superior division |  | 9.77 | -38 | -80 | 36 |
|  | **Cluster 4** | 4,400 |  |  |  |  |
|  | R Postcentral Gyrus |  | 9.34 | 66 | -10 | 30 |
|  | R Precentral Gyrus |  | 8.55 | 60 | -6 | 42 |
|  | **Cluster 5** | 2,104 |  |  |  |  |
|  | L Frontal Medial Cortex |  | 6.63 | -4 | 52 | -8 |
|  | R Frontal Pole |  | 9.06 | 0 | 64 | -10 |
|  | R Frontal Medial Cortex |  | 7.28 | 2 | 50 | -12 |
|  | **Cluster 6** | 1,904 |  |  |  |  |
|  | R Hippocampus |  | 11.81 | 22 | -20 | -18 |
|  | **Cluster 7** | 1,448 |  |  |  |  |
|  | L Hippocampus |  | 10.29 | -22 | -20 | -16 |
|  | L Amygdala |  | 5.86 | -12 | -6 | -20 |
|  | **Cluster 8** | 1,168 |  |  |  |  |
|  | L Central Opercular Cortex |  | 9.57 | -38 | -12 | 18 |
|  | L Insular Cortex |  | 7.60 | -40 | -8 | 6 |
|  | **Cluster 9** | 1,048 |  |  |  |  |
|  | L Parahippocampal Gyrus, posterior division |  | 7.98 | -24 | -38 | -16 |
|  | L Temporal Fusiform Cortex, posterior division |  | 7.86 | -34 | -34 | -18 |
|  | **Cluster 10** | 856 |  |  |  |  |
|  | R Lingual Gyrus |  | 9.44 | 26 | -38 | -12 |
|  | R Parahippocampal Gyrus, posterior division |  | 8.43 | 26 | -34 | -16 |
|  | R Temporal Fusiform Cortex, posterior division |  | 8.13 | 34 | -36 | -14 |
|  | **Cluster 11** | 656 |  |  |  |  |
|  | R Superior Frontal Gyrus |  | 7.01 | 22 | 30 | 44 |
|  | **Cluster 12** | 576 |  |  |  |  |
|  | R Insular Cortex |  | 9.14 | 38 | -8 | 12 |
|  | R Central Opercular Cortex |  | 5.85 | 46 | -10 | 16 |
|  | **Cluster 13** | 536 |  |  |  |  |
|  | R Middle Temporal Gyrus, anterior division |  | 6.69 | 60 | -4 | -16 |
|  | **Cluster 14** | 376 |  |  |  |  |
|  | R Subcallosal Cortex |  | 8.48 | 2 | 12 | -4 |
|  | **Cluster 15** | 272 |  |  |  |  |
|  | R Lateral Occipital Cortex, superior division |  | 6.03 | 20 | -66 | 50 |
|  | **Cluster 16** | 136 |  |  |  |  |
|  | L Juxtapositional Lobule Cortex |  | 7.66 | -4 | 0 | 64 |
|  | **Cluster 17** | 120 |  |  |  |  |
|  | R Postcentral Gyrus |  | 6.09 | 48 | -16 | 52 |
|  | **Cluster 18** | 112 |  |  |  |  |
|  | L Middle Temporal Gyrus, temporooccipital part |  | 6.07 | -60 | -62 | -8 |
|  | L Lateral Occipital Cortex, inferior division |  | 5.57 | -58 | -68 | -8 |
|  | **Cluster 19** | 96 |  |  |  |  |
|  | L Superior Frontal Gyrus |  | 5.67 | -22 | 26 | 46 |
|  | **Cluster 20** | 88 |  |  |  |  |
|  | L Precuneus Cortex |  | 5.52 | -2 | -60 | 64 |
|  | **Cluster 21** | 64 |  |  |  |  |
|  | R Frontal Pole |  | 6.26 | 0 | 62 | 6 |
|  | **Cluster 22** | 48 |  |  |  |  |
|  | R Frontal Pole |  | 5.50 | 10 | 70 | 12 |
|  | **Cluster 23** | 48 |  |  |  |  |
|  | L Lateral Occipital Cortex, superior division |  | 5.76 | -8 | -86 | 42 |
|  | **Cluster 24** | 40 |  |  |  |  |
|  | L Lateral Occipital Cortex, superior division |  | 5.42 | -16 | -84 | 48 |
|  | **Cluster 25** | 32 |  |  |  |  |
|  | R Accumbens |  | 6.01 | 12 | 14 | -8 |
|  | **Cluster 26** | 32 |  |  |  |  |
|  | R Caudate |  | 6.12 | 6 | 10 | -2 |
|  | **Cluster 27** | 32 |  |  |  |  |
|  | R Precuneus Cortex |  | 5.50 | 12 | -50 | 42 |
|  | **Cluster 28** | 24 |  |  |  |  |
|  | L Middle Temporal Gyrus, anterior division |  | 5.12 | -62 | -4 | -16 |
|  | **Cluster 29** | 24 |  |  |  |  |
|  | L Frontal Pole |  | 5.78 | -20 | 66 | 12 |
|  | **Cluster 30** | 24 |  |  |  |  |
|  | L Planum Temporale |  | 5.37 | -48 | -38 | 16 |
|  | **Cluster 31** | 24 |  |  |  |  |
|  | L Supramarginal Gyrus, posterior division |  | 5.38 | -56 | -42 | 18 |
|  | **Cluster 32** | 24 |  |  |  |  |
|  | L Frontal Pole |  | 5.65 | -10 | 70 | 18 |
|  | **Cluster 33** | 24 |  |  |  |  |
|  | L Superior Parietal Lobule |  | 5.20 | -28 | -54 | 52 |
|  | **Cluster 34** | 16 |  |  |  |  |
|  | R Frontal Medial Cortex |  | 5.29 | 2 | 40 | -18 |
|  | **Cluster 35** | 16 |  |  |  |  |
|  | L Lateral Occipital Cortex, superior division |  | 5.17 | -6 | -72 | 58 |
|  | **Cluster 36** | 8 |  |  |  |  |
|  | L Amygdala |  | 5.31 | -30 | 2 | -18 |
|  | **Cluster 37** | 8 |  |  |  |  |
|  | R Frontal Pole |  | 5.17 | 36 | 38 | -8 |
|  | **Cluster 38** | 8 |  |  |  |  |
|  | L Lateral Occipital Cortex, superior division |  | 5.28 | -30 | -76 | 48 |
|  | **Cluster 39** | 8 |  |  |  |  |
|  | R Lateral Occipital Cortex, superior division |  | 5.37 | 20 | -64 | 60 |

**SUPPLEMENTARY REFERENCES**

Avery, S. N., Clauss, J. A., & Blackford, J. U. (2016). The human BNST: Functional role in anxiety and addiction. *Neuropsychopharmacology*, *41*, 126-141. [https://doi.org/10.1038/npp.2015.185](https://doi.org/10.1038/npp.2015.185) 

Daniel-Watanabe, L., & Fletcher, P. C. (2022). Are fear and anxiety truly distinct? *Biological Psychiatry Global Open Science*, *2*, 341-349. [https://doi.org/https://doi.org/10.1016/j.bpsgos.2021.09.006](https://doi.org/https://doi.org/10.1016/j.bpsgos.2021.09.006) 

Davis, M., Walker, D. L., Miles, L., & Grillon, C. (2010). Phasic vs sustained fear in rats and humans: Role of the extended amygdala in fear vs anxiety. *Neuropsychopharmacology*, *35*, 105-135. [https://doi.org/10.1038/npp.2009.109](https://doi.org/10.1038/npp.2009.109) 

Domschke, K. (2022). Fear and anxiety-Distinct or "kindred" phenomena? *Biol Psychiatry Glob Open Sci*, *2*, 314-315. [https://doi.org/10.1016/j.bpsgos.2022.07.001](https://doi.org/10.1016/j.bpsgos.2022.07.001) 

Fox, A. S., & Shackman, A. J. (2019). The central extended amygdala in fear and anxiety: Closing the gap between mechanistic and neuroimaging research. *Neuroscience letters*, *693*, 58-67. [https://doi.org/https://doi.org/10.1016/j.neulet.2017.11.056](https://doi.org/https://doi.org/10.1016/j.neulet.2017.11.056) 

Grogans, S. E., Bliss-Moreau, E., Buss, K. A., Clark, L. A., Fox, A. S., Keltner, D., . . . Shackman, A. J. (2023). The nature and neurobiology of fear and anxiety: State of the science and opportunities for accelerating discovery. *Neuroscience & Biobehavioral Reviews*, *151*, 105237. [https://doi.org/https://doi.org/10.1016/j.neubiorev.2023.105237](https://doi.org/https://doi.org/10.1016/j.neubiorev.2023.105237) 

Grupe, D. W., & Nitschke, J. B. (2013). Uncertainty and anticipation in anxiety: an integrated neurobiological and psychological perspective. *Nat Rev Neurosci*, *14*, 488-501. [https://doi.org/nrn3524](https://doi.org/nrn3524) [pii]

10.1038/nrn3524 

Gungor, N. Z., & Paré, D. (2016). Functional heterogeneity in the bed nucleus of the stria terminalis. *Journal of Neuroscience*, *36*, 8038-8049. 

Lebow, M. A., & Chen, A. (2016). Overshadowed by the amygdala: the bed nucleus of the stria terminalis emerges as key to psychiatric disorders. *Mol Psychiatry*, *21*, 450-463. [https://doi.org/10.1038/mp.2016.1](https://doi.org/10.1038/mp.2016.1) 

LeDoux, J. E., & Pine, D. S. (2016). Using neuroscience to help understand fear and anxiety: A two-system framework. *Am J Psychiatry*, *173*, 1083-1093. [https://doi.org/10.1176/appi.ajp.2016.16030353](https://doi.org/10.1176/appi.ajp.2016.16030353) 

Mobbs, D., Headley, D. B., Ding, W., & Dayan, P. (2020). Space, time, and fear: Survival computations along defensive circuits. *Trends in cognitive sciences*, *24*, 228-241. [https://doi.org/10.1016/j.tics.2019.12.016](https://doi.org/10.1016/j.tics.2019.12.016) 

Moscarello, J. M., & Penzo, M. A. (2022). The central nucleus of the amygdala and the construction of defensive modes across the threat-imminence continuum. *Nat Neurosci*, *25*, 999-1008. [https://doi.org/10.1038/s41593-022-01130-5](https://doi.org/10.1038/s41593-022-01130-5) 

NIMH. (2011). *Negative valence systems: Workshop proceedings (March 13, 2011 – March 15, 2011; Rockville, Maryland)*. Retrieved July 1 from [https://www.nimh.nih.gov/research/research-funded-by-nimh/rdoc/negative-valence-systems-workshop-proceedings.shtml](https://www.nimh.nih.gov/research/research-funded-by-nimh/rdoc/negative-valence-systems-workshop-proceedings.shtml)

Perusini, J. N., & Fanselow, M. S. (2015). Neurobehavioral perspectives on the distinction between fear and anxiety. *Learn Mem*, *22*, 417-425. [https://doi.org/10.1101/lm.039180.115](https://doi.org/10.1101/lm.039180.115) 

Shackman, A. J., & Fox, A. S. (2016). Contributions of the central extended amygdala to fear and anxiety. *Journal of Neuroscience*, *36*, 8050-8063. [https://doi.org/10.1523/JNEUROSCI.0982-16.2016](https://doi.org/10.1523/JNEUROSCI.0982-16.2016) 

Shackman, A. J., Tromp, D. P. M., Stockbridge, M. D., Kaplan, C. M., Tillman, R. M., & Fox, A. S. (2016). Dispositional negativity: An integrative psychological and neurobiological perspective. *Psychological Bulletin*, *142*, 1275-1314. 

Tovote, P., Fadok, J. P., & Lüthi, A. (2015). Neuronal circuits for fear and anxiety. *Nat Rev Neurosci*, *16*, 317-331. [https://doi.org/10.1038/nrn3945](https://doi.org/10.1038/nrn3945)
